# Centrosomal and ciliary targeting of CCDC66 requires cooperative action of centriolar satellites, microtubules and molecular motors

**DOI:** 10.1038/s41598-019-50530-4

**Published:** 2019-10-03

**Authors:** Deniz Conkar, Halil Bayraktar, Elif Nur Firat-Karalar

**Affiliations:** 1 Department of Molecular Biology and Genetics, Koç University, Istanbul, 34450 Turkey; 2 Department of Molecular Biology and Genetics, Istanbul Technical University, Istanbul, 34450 Turkey

**Keywords:** Centrosome, Cilia

## Abstract

Mammalian centrosomes and cilia play key roles in many cellular processes and their deregulation is linked to cancer and ciliopathies. Spatiotemporal regulation of their biogenesis and function in response to physiological stimuli requires timely protein targeting. This can occur by different pathways, including microtubule-dependent active transport and via centriolar satellites, which are key regulators of cilia assembly and signaling. How satellites mediate their functions and their relationship with other targeting pathways is currently unclear. To address this, we studied retinal degeneration gene product CCDC66, which localizes to centrosomes, cilia, satellites and microtubules and functions in ciliogenesis. FRAP experiments showed that its centrosomal pool was dynamic and the ciliary pool associated with the ciliary axoneme and was stable. Centrosomal CCDC66 abundance and dynamics required microtubule-dependent active transport and tethering, and was inhibited by sequestration at satellites. Systematic quantitation of satellite dynamics identified only a small fraction to display microtubule-based bimodal motility, consistent with trafficking function. Majority displayed diffusive motility with unimodal persistence, supporting sequestration function. Together, our findings reveal new mechanisms of communication between membrane-less compartments.

## Introduction

The mammalian centrosome/cilium complex is composed of the centrosome, the cilium and the centriolar satellites, which together function in a diverse set of cellular processes ranging from cell division to cellular signaling. Centrosomes and cilia are both microtubule-based structures that are organized into structurally distinct domains. Their biogenesis, maintenance and function are tightly regulated in response to physiological stimuli including cell cycle cues^1,2^. In G0, most animal cells have one centrosome that is composed of a pair of centrioles. During the G1/S-phase transition, centrioles duplicate to form procentrioles, which elongate throughout S and G2 phases. The mitotic spindle then segregates the duplicated centrioles to ensure that each daughter cell receives a pair of centrioles. In quiescent cells, the older of the centrioles, the mother centriole, docks to the plasma membrane to form the primary cilium, which is a nexus for developmentally important signaling pathways including Hedgehog signaling. Defects in the structure and function of the centrosome/cilium complex are linked to various human diseases including ciliopathies and cancer^2–4^.

Bioinformatic, genomic, transcriptomic and proteomic studies have identified over 100 proteins that localize to the centrosomes and cilia^5–13^. Dynamic regulation of the biogenesis, maintenance and function of the centrosome/cilium complex in response to physiological stimuli requires timely and efficient targeting of proteins^14–16^. What makes such spatiotemporal regulation complex is the membrane-less nature of the centrosome and the semiclosed nature of the primary cilium. Although they are not membrane-bound compartments, both centrosomes and cilia are organized into structurally distinct domains^17–19^. Moreover, some proteins, like gamma-tubulin, are less abundant at the centrosome/cilium than in the cytoplasm^20^. A major unresolved question that pertains to our understanding of centrosomal and ciliary regulation relates to dissecting the pathways that target proteins to centrosomes and cilia at the right time and place, as well as that limit their recruitment from cytoplasmic pools.

The abundance and dynamic localization of proteins at the centrosomes and cilia can be regulated by delivery, retention, and/or removal. For cilia, key regulators of protein content are the vesicular and intraciliary trafficking pathways as well as gating mechanisms such as the transition zone and the septin-based diffusion barrier^21,22^. For centrosomal protein targeting, among the pathways reported are those dependent on the microtubule cytoskeleton, molecular motors, centriolar satellites, diffusion and mRNA localization^23–30^. Passive diffusion by itself is sufficient for targeting all or a fraction of some proteins to the centrosome. For example, the dynamic exchange of centrosomal gamma-tubulin with the cytoplasm does not require microtubules^31^. However, passive diffusion is limited for the directional and long-range targeting of proteins, which is essential for the timely and rapid changes in composition that occurs during the cell cycle and development. This limitation is overcome in part by microtubule and molecular motor-dependent active transport, which was reported for centrosomal targeting of CDK5RAP2^32,33^, Par6alpha^34^, pericentrin, and NUMA^30,35,36^. The underlying mechanisms of this microtubule-based active transport, as well as its relationship with other transport pathways, in particular how they cooperate or compete with each other in specific cellular contexts and in response to different signals, is not known.

Centriolar satellites are an array of granules that cluster around the centrosome/cilium-complex in a microtubule- and dynein-dependent manner^28,37^. They are scaffolded by Pericentriolar Material-1 (PCM1), which is essential for their integrity and mediates their interaction with a wide range of centrosome and cilium proteins^15,38^. Satellites are ubiquitous in vertebrate cells and have emerged as key regulators of the biogenesis and function of the centrosome/cilium complex. Although they are widely accepted to mediate their functions through regulating protein targeting, the underlying mechanisms of this regulation remains unresolved. The motility of satellites towards and away from the centrosome along microtubules, and the decrease in the centrosomal levels of several proteins such as pericentrin, centrin and ninein in PCM1-depleted satellite-less cells have suggested a possible active transport function for satellites^37,39,40^. According to this model, satellites move along microtubules in a molecular motor-dependent manner to deliver and remove proteins at the centrosome. However, the absence of quantitative analysis of satellite dynamics and direct evidence showing their role in protein targeting makes this model incomplete. Moreover, recent studies showed that satellites regulate cilium formation by limiting the incorporation of the ubiquitin ligase Mib1 and BBS4 at the centrosome and cellular stress response by sequestration of Cep131 and other satellite proteins^41^. This indicates a possible sequestration function^38,42^. To elucidate the molecular mechanism of how satellites regulate protein targeting and their relationship to microtubules and molecular motors, we focused on studying the dynamic compartmentalization of CCDC66 within the centrosome/cilium-complex.

CCDC66 is a component of the centrosome/cilium complex and its mutations in dogs and mouse are linked to retinal degeneration^43–45^. In interphase cells, it localizes to the centrosome and centriolar satellites. As cells enter mitosis, it accumulates at spindle poles and spindle microtubules with concomitant dissolution of its satellite pool that reassembles at the end of mitosis. Upon serum starvation, CCDC66 also localizes to the primary cilium, analogous to BBS4 of the BBSome, which regulates ciliary protein trafficking^43^. The multitude of cellular pools of CCDC66 is paralleled by its various functions in cilium assembly, ciliary recruitment of the BBSome complex, and spindle pole and centriolar satellite organization^43,46^. Importantly, the retinal degeneration mutation disrupts the centrosomal and ciliary localization of CCDC66 and its interactions^43^.

How CCDC66 dynamically compartmentalizes between its different cellular pools is not known. Understanding the mechanism underlying this compartmentalization is required to gain insight into regulation of its various functions and the cellular defects that lead to CCDC66-linked retinal degeneration. To address these questions, we developed imaging assays to quantify the dynamic behavior of CCDC66 at the centrosomes, satellites and cilia and showed that CCDC66 has different dynamics at these compartments. We then elucidated the interplay between satellites, microtubules and molecular motors in CCDC66 centrosomal and ciliary targeting by employing these assays in cells selectively disrupted for satellite integrity and distribution, or microtubule-dependent active transport, organization and stability. While satellites inhibited the centrosomal and ciliary recruitment of CCDC66, microtubules and motors facilitated its dynamic localization at the centrosome. In addition, systematic quantification of the dynamic behavior of satellites showed that the majority displayed random diffusive motility, while the rest were persistently motile towards or away from the centrosome. This suggests that only a small percentage of satellites might mediate trafficking at a given time. Together, our results identify “sequestration” as another layer of regulation of protein targeting by satellites, in addition to trafficking-based mechanisms, and provide insight into cellular compartmentalization through membrane-less structures.

## Results

### Centriolar satellites negatively regulate CCDC66 centrosomal recruitment and kinetics

**Figure 1:**
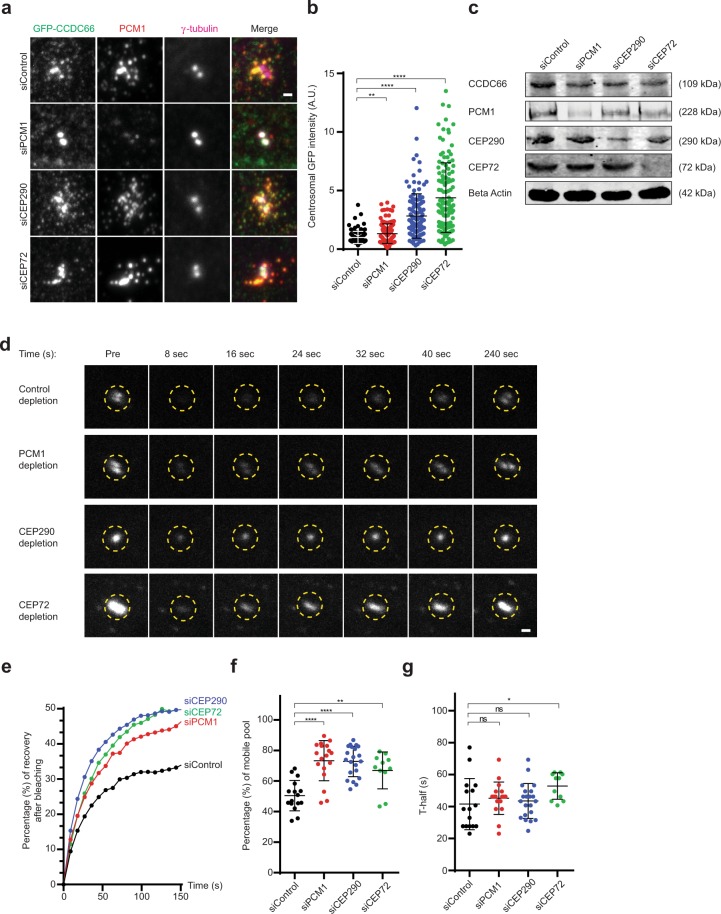
Centriolar satellites inhibit CCDC66 dynamic localization at the centrosome. (**a**) Effect of PCM1, CEP72 and CEP290 depletion on CCDC66 level at the centrosome. RPE1::GFP-CCDC66 cells were transfected with control, PCM1, CEP290 or CEP72 siRNAs for 48 h. Cells were then fixed and stained for GFP, PCM1 and gamma tubulin. Images represent centrosomes in cells from the same coverslip taken with the same camera settings. Scale bar, 1 μm. (**b**) Quantification of (**a**). GFP-CCDC66 fluorescence intensities were measured in a 2.5 μm^2^ circular area around the centrosome from two independent experiments. Levels are normalized to the mean of the control group (=1). n )0.50 cells for each group. t-test was used for statistical analysis. Standard error of mean (SEM): siControl = 0.046, siPCM1 = 0.075, siCEP290 = 0.17, siCEP72 = 0.27. (**c**) Effect of PCM1, CEP72 and CEP290 depletion on total cellular CCDC66 levels. Cells were transfected with control, PCM1, CEP290 or CEP72 siRNAs for 48 h. Cell extracts were cells were immunoblotted for CCDC66, PCM1, CEP290 and CEP72. Beta-actin was used as a loading control. (**d**) Effect of PCM1, CEP72 and CEP290 depletion on CCDC66 dynamics at the centrosome. RPE1::GFP-CCDC66 cells were transfected with control, PCM1, CEP290 or CEP72 siRNAs for 48 h. 2.5 μm^2^ circular area around the centrosome marked by yellow dashed circle was photobleached and imaged for 250 seconds after photobleaching. Still images represent centrosomal GFP-CCDC66 signal at the indicated times. Scale bar, 1 μm. (**e**) Percentage of recovery graph from (**d**). Individual FRAP experiments from two independent experiments were fitted into one phase association curves. n = 8 for control and PCM1, n = 8 for CEP290 and n = 6 for CEP72 depleted cells per group. Half-time of and mobile pool were calculated using recovery data. (**f**) mobile pool of (**d**). Error bars, SEM: siControl = 2.49, siPCM1 = 3.10, siCEP290 = 2.16, siCEP72 = 3.64 and (**g**) half-time analysis of (**d**). Error bars, SEM: siControl = 4.00, siPCM1 = 2.39, siCEP290 = 2.89, siCEP72 = 2.50.

The localization of CCDC66 to centriolar satellites and its physical and proximity interactions with satellite proteins suggest that satellites play important roles in CCDC66 function and regulation. Centriolar satellites have been implicated in both active transport and in sequestration of centrosome and cilium proteins^28,37,47^. To distinguish between these models, we quantified the centrosomal levels and dynamics of CCDC66 in control and PCM1-depleted satellite-less cells. In these experiments, we used the human retinal pigmental epithelial (RPE1) cell line stably expressing GFP-CCDC66 at near-endogenous levels^43^. Centrosomal levels of GFP-CCDC66 in PCM1-depleted cells (1.32 ± 0.07, p = 0.0162) significantly increased relative to control cells (siControl =1.00 ± 0.05) (Fig. 1a,b). Depletion of PCM1 did not change the total levels of CCDC66 (Fig. 1c), suggesting that satellites regulate CCDC66 targeting to centrosomes. To determine the consequences of satellite loss on the dynamics of centrosomal recruitment, we performed fluorescence recovery after photobleaching (FRAP) experiments in control (n nF16 in total) and PCM1-depleted cells (n = 16 in total) (Figs 1d, S1A,B). In control cells, 50.4% ± 2.49 of centrosomal GFP-CCDC66 recovered rapidly with a half time of 40 s ± 4.00, identifying this as a dynamic pool with the rest being stably incorporated at the centrosome (Fig. 1e–g). In PCM1-depleted cells, the percentage of recovery significantly increased (73.3% ± 3.10, p < 0.0001) but half time (45.2 s ± 2.40) did not change (Fig. 1e–g). Of note, depletion of PCM1 resulted in a decrease in the percentage of cells with radial microtubule arrays (Fig. S1C)^39^. Together, these results identify an inhibitory role for satellites in centrosomal recruitment of CCDC66. However, they do not distinguish whether this regulation is mediated by removal of CCDC66 from the centrosome or its sequestration at satellites.

In previous work, we identified interactions between CCDC66 and several centriolar satellite proteins: a physical interaction with PCM1 and CEP290 was identified using co-immunoprecipitation experiments, and a proximity interaction with CEP72 using Biotin Identification (BioID) proximity labeling experiments^43^. The satellite proteins CEP72 and CEP290 are required for cilium assembly and ciliary content regulation, and their depletion results in satellite accumulation around the centrosome^42,48,49^. To investigate the role of these interactions and the consequences of satellite clustering on CCDC66 targeting, we quantified the abundance and dynamics of centrosomal CCDC66 in CEP290- and CEP72-depleted cells (Fig. S1D,E). A significant increase in the centrosomal abundance of CCDC66 relative to control cells (siControl = 1.00 ± 0.05) was observed for cells depleted of either CEP290 (2.79 ± 0.17, p < 0.0001) or CEP72 (4.38 ± 0.27, p < 0.0001, n = 120 in total) (Fig. 1a,b). Likewise, a significant increase in the percentage of recovery of CCDC66 at centrosomes relative to control cells (50.4% ± 2.49) was observed for cells depleted of either CEP290 (72.8% ± 2.16, p < 0.0001, n = 17) or CEP72 (66.9% ± 3.64, p = 0.0038, n = 12 in total) (Figs 1d–f, S1F,G). While there was no change in the rate of recovery for cells depleted of CEP290 (43.5 s ± 2.39), a significant increase in halftime was observed in cells depleted of CEP72 (52.9 s ± 2.50, p = 0.0323) compared to control cells (41.5 s ± 4.00) (Fig. 1g). Notably, the changes in the recovery percentages were comparable to the ones in PCM1-depleted cells. Collectively, these results demonstrate that disruption of satellite function and distribution by depleting CEP72 and CEP290, as well as loss of satellites by depleting PCM1, facilitates centrosomal recruitment and dynamics of CCDC66. This suggests that these proteins cooperate at satellites to regulate CCDC66 centrosomal targeting.

### Centrosomal CCDC66 recruitment requires an intact and dynamic microtubule network

**Figure 2:**
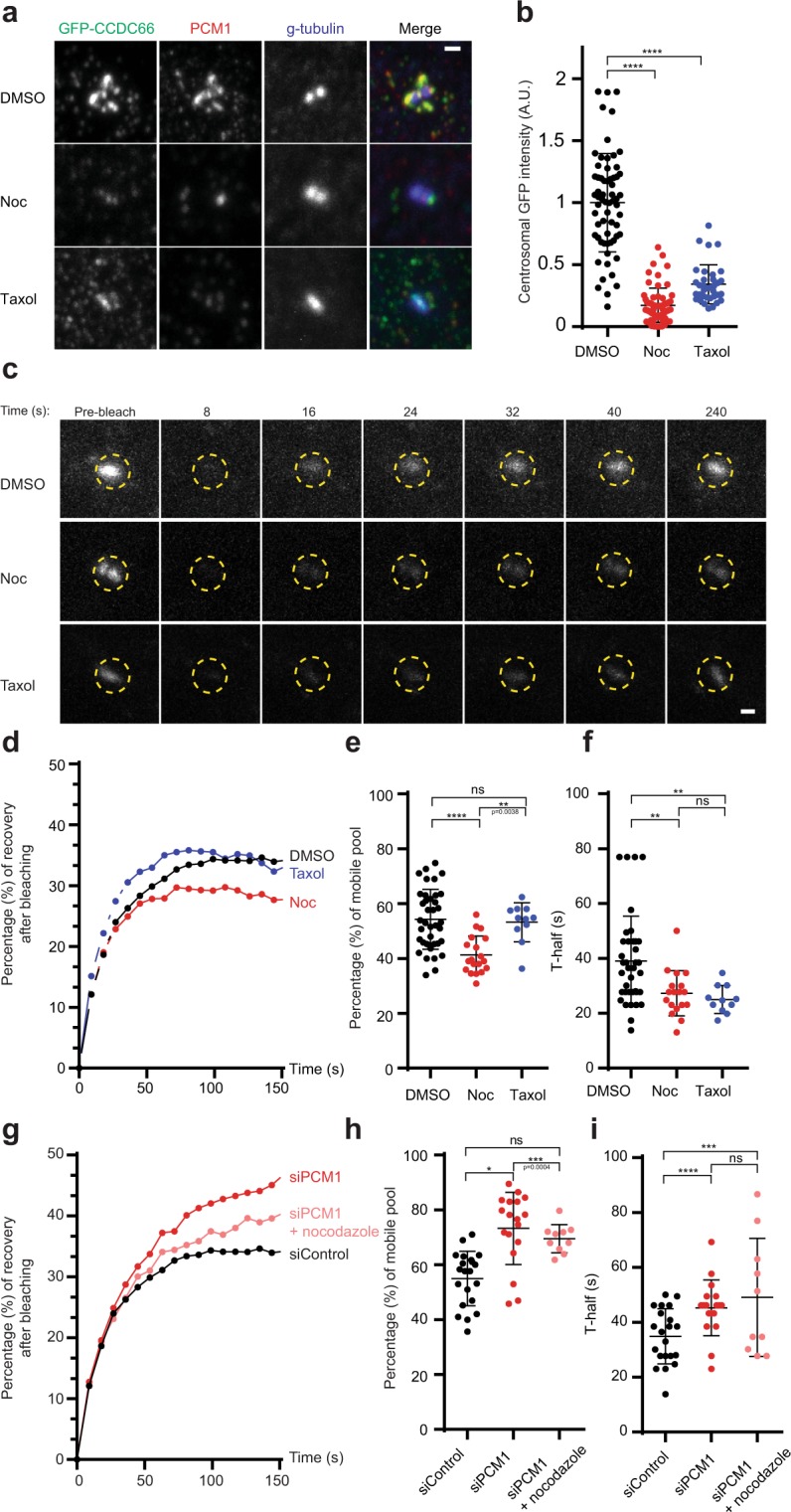
An intact and dynamic microtubule network is required for CCDC66 dynamic localization at the centrosome. (**a**) Effect of microtubule depolymerization and stabilization on CCDC66 level at the centrosome. RPE1::GFP-CCDC66 cells were treated with 0.1% DMSO, 5 μg/ml nocodazole or 5 μM taxol for 1 h. Cells were then fixed and stained for GFP, PCM1 and gamma tubulin. Images represent centrosomes in cells from the same coverslip taken with the same camera settings. Scale bar, 1 μm. (**b**) Quantification of (**a**). GFP-CCDC66 fluorescence intensities were measured in a 2.5 μm^2^ circular area around the centrosome from two independent experiments. Levels are normalized to the mean of the control group ( =1). n = 50 cells for each group. t-test was used for statistical analysis. Error bars, SEM: DMSO control = 0.05, nocodazole = 0.02, taxol = 0.03. **<00.05, ***0.0005. (**c**) Effect of microtubule depolymerization and stabilization on CCDC66 dynamics at the centrosome. RPE1::GFP-CCDC66 cells were treated with 0.1% DMSO, 5 μg/ml nocodazole or 5 μM taxol for 1 h. 2.5 μm^2^ circular area around the centrosome marked by yellow dashed circle was photobleached and imaged for 250 seconds after photobleaching. Still images represent centrosomal GFP-CCDC66 signal at the indicated times. Scale bar, 1 μm. (**d**) Percentage of recovery graph from (**c**). Individual FRAP experiments from two independent experiments were fitted into one phase association curves. n = 10 for DMSO, n = 9 for nocodazole and n = 5 for taxol treated cells per group. Half-time of and mobile pool were calculated using recovery data. (**e**) mobile pool of (**d**). Error bars, SEM: DMSO = 1.77, nocodazole = 1.57, taxol = 2.13. (**f**) half-time analysis of (**d**). Error bars, SEM: DMSO = 2.67, nocodazole = 1.95, taxol = 1.52. (**g**) Combinatorial effect of microtubule depolymerization and PCM1 depletion on CCDC66 dynamics at the centrosome. RPE1::GFP-CCDC66 cells were transfected with control and PCM1 siRNAs for 48 h and then treated with 5 μg/ml nocodazole for 1 h. Individual FRAP experiments from two independent experiments were fitted into one phase association curves and percentage of recovery graphs were generated. n = 12 for control depleted, n = 12 for PCM1 depleted and n = 10 for PCM1 depleted and nocodazole treated cells per group. Half-time of and mobile pool were calculated using recovery data. (**h**) mobile pool of (d) Error bars, SEM: siControl = 2.19, siPCM1 = 1.48, siPCM1+nocodazole = 1.63. (**i**) half-time analysis of (d). Error bars, SEM: siControl = 2.2, siPCM1 = 2.4, siPCM1 = nocodazole = 6.80.

The clustered localization of satellites around the centrosomes requires an intact microtubule network^37^. Using *in vitro* and *in vivo* studies, we previously showed that CCDC66 localizes to microtubules and directly interacts with them^43^. Based on these lines of evidence, we hypothesized that microtubules might regulate CCDC66 targeting either by maintaining satellite proximity to the centrosome for fast exchange of material, tethering CCDC66 at the centrosome by generating binding sites, and/or actively transporting CCDC66 to centrosomes. To test these models, we quantified centrosomal CCDC66 abundance and dynamics in RPE1::GFP-CCDC66 cells treated with either nocodazole to depolymerize microtubules, or taxol to stabilize microtubules. Both drug treatments result in loss of centrosome-nucleated microtubules^50,51^ and a consequent declustering of satellites throughout the cytoplasm (Fig. S2A–C). In contrast to the phenotypes of satellite-less cells, both treatments resulted in a significant decrease in the centrosomal levels of CCDC66 (DMSO control = 1 ± 0.05; nocodazole = 0.17 ± 0.02, p < 0.0001; taxol = 0.34 ± 0.03, p < 0.0001, n = 70 in total) (Fig. 2a,b). FRAP analysis of centrosomal GFP-CCDC66 showed both nocodazole and taxol treated cells had significantly faster recovery rates (DMSO control = 39 s ± 2.67, n = 20; nocodazole = 27.3 s ± 1.95, p = 0.0090, n = 18; taxol = 25 s ± 1.52, p = 0.0090, n = 10 in total) (Figs 2c,d,f, Fig. S2D–F). There was a significant decrease in the percentage of recovery in nocodazole-treated cells but not in taxol-treated cells (DMSO control = 54.2% ± 1.77; nocodazole = 41.3% ± 1.57, p < 0.0001; taxol = 53.2% ± 2.13, p = 0.9431) (Fig. 2c–e). To examine the relationship between satellites and microtubules for protein targeting to centrosomes, FRAP experiments on centrosomal GFP-CCDC66 were performed in cells depleted for PCM1 and treated with nocodazole. In PCM1-depleted cells, depolymerization of microtubules resulted in a significant decrease in the centrosomal abundance of CCDC66 and its percentage of recovery in FRAP experiments (Control depleted cells: mobile pool = 55.4% ± 2.19, halftime = 34.9 s ± 2.20, PCM1-depleted cells: mobile pool = 80.8% ± 1.48, halftime = 45.2 s ± 2.39, p < 0.0001, nocodozole-treated PCM1-depleted cells: mobile pool = 69.50% ± 1.63, halftime = 49.1 s ± 6.79, p < 0.0001) (Figs 2g–i, S2G–I). These results reveal that microtubules are required for CCDC66 centrosomal abundance and dynamic localization, and that satellites have an inhibitory role in this process.

### The dynein complex interacts with CCDC66 and mediates centrosomal recruitment and dynamics of CCDC66

**Figure 3:**
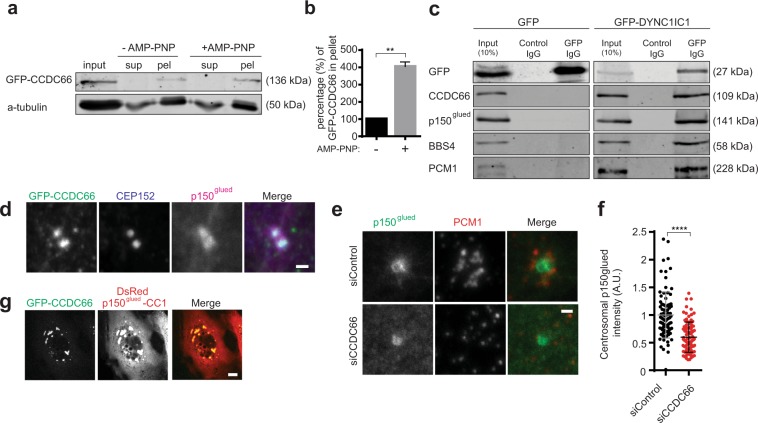
They dynein complex interacts and co-localizes with CCDC66. (**a**) Analysis of CCDC66 microtubule association in cells treated with unhydrolyzable ATP analog AMP-PNP. HEK293T cells were transfected with GFP-CCDC66, treated with 0.5 mM unhydrolyzable ATP analog AMP-PNP and *in vitro* microtubule pelleting experiments were performed with extracts from control and AMP-PNP-treated cells. Equal volumes of input, supernatant and pellet fractions were immunoblotted for GFP and alpha-tubulin. Graph represents the mean values from two independently performed pelleting experiments, p = 0.0038. (**b**) Quantification of GFP-CCDC66 amount in pellet from (**a**). Percentage of GFP-CCDC66 in the pellet relative to input was quantified by measuring GFP band intensities of pellet and input fractions and normalizing them to alpha tubulin levels. (**c**) Immunoprecipitation of the dynein complex. HEK293T cells were transfected with GFP or GFP-DYNC1IC1 for 48 h. Complexes were immunoprecipitated (IP) with control IgG or anti-GFP antibody, and co-precipitated proteins were detected with GFP, CCDC66, p150^glued^, BBS4 and PCM1. (**d**) Localization of CCDC66 and p150^glued^ at the centrosome. RPE1::GFP-CCDC66 cells were fixed with methanol and stained with GFP, p150^glued^ and CEP152 (centrosome marker). Scale bar, 1 μm. (**e**) Effect of CCDC66 depletion on p150^glued^ level at the centrosome. RPE1 cells were transfected with control and CCDC66 siRNAs for 48 h. Cells were then fixed and stained for p150^glued^ and PCM1. Images represent centrosomes in cells from the same coverslip taken with the same camera settings. Scale bar, 1 μm. (**f**) Quantification of (e). p150^glued^ fluorescence intensities were measured in a 2.5 μm^2^ circular area around the centrosome from two independent experiments. Levels are normalized to the mean of the control group (=1). n = 50 cells for each group. t-test was used for statistical analysis. Error bars, SEM: siControl = 0.04, siCCDC66 = 0.03. (**g**) Effects of p150^glued^-CC overexpression on localization of CCDC66. RPE1::GFP-CCDC66 were transfected with DsRed p150^glued^ 217-548 for 24 h, fiexed and stained for GFP. Scale bar, 10 μm.

The cytoplasmic dynein motor transports its cargo along microtubules by associating with the dynactin complex. Among its cargoes are many centrosome proteins^52^. A recent BioID-based human cytoplasmic dynein complex interactome identified CCDC66 as a proximity partner of the dynein adaptors BICD2 and NINL^53^. To examine whether the interaction between microtubules and CCDC66 requires ATPase-dependent motor activity, cells expressing GFP-CCDC66 were treated with the ATP analog AMP-PNP, which enhances the affinity of molecular motors to microtubules at low concentrations^54,55^. *In vitro* microtubule pelleting experiments from control and 0.5 mM AMP-PNP-treated cells revealed that the fraction of GFP-CCDC66 that co-pelleted with microtubules was significantly higher in the presence of AMP-PNP relative to control (p = 0.0038), suggesting that molecular motors directly or indirectly mediate the association of CCDC66 with microtubules (Fig. 3a,b). We then tested whether dynein mediates this interaction by performing immunoprecipitation experiments in HEK293T cells expressing GFP-DYNC1IC1. CCDC66 along with BBS4, the dynactin complex subunit p150^glued^ and PCM1 co-pelleted with GFP-DYNC1lC1, but not with GFP (Fig. 3c). In agreement with this interaction, GFP-CCDC66 co-localized with p150^glued^ at the centrosome (Fig. 3d) and CCDC66 depletion resulted in a significant decrease in the centrosomal levels of p150^glued^ (siControl = 1.00 ± 0.04; siCCDC66 = 0.6 ± 0.03, p < 0.0001, n = 100 in total) relative to control (Fig. 3e,f). Likewise, overexpression of the dynein-binding fragment of the dynactin subunit, DsRed-p150^glued^ 217–548 aa (p150^glued^-CC1)^56^, disrupted the centriolar satellite organization and resulted in formation of cytoplasmic aggregates that sequestered GFP-CCDC66 (Fig. 3g). Together, these results reveal a cellular complex between CCDC66, dynein/dynactin, satellites and microtubules.

**Figure 4:**
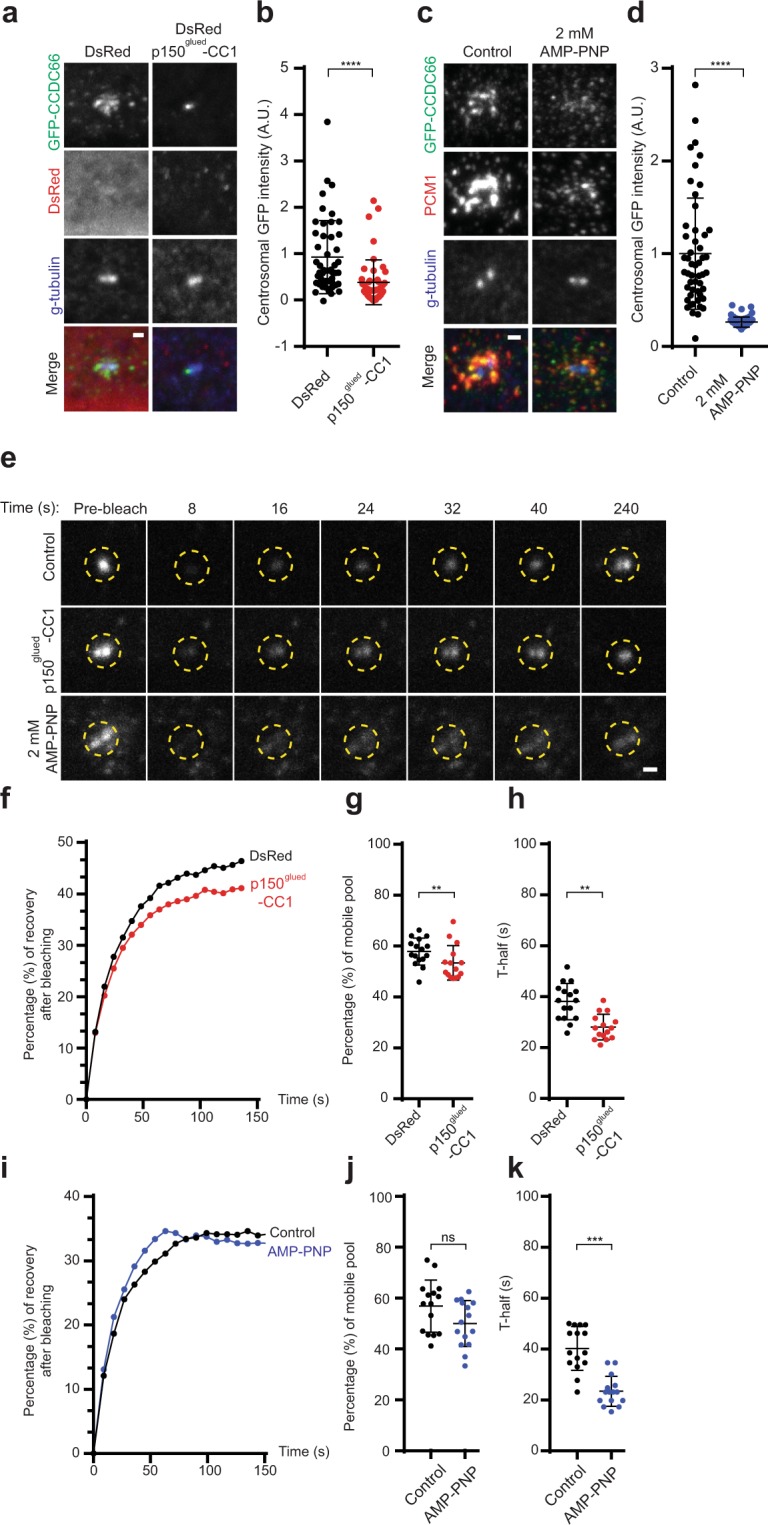
Activity of molecular motors are required for CCDC66 dynamic localization at the centrosome. (**a**) Effect of inhibiting dynein complex activity on CCDC66 level at the centrosome. RPE1::GFP-CCDC66 cells were transfected with DsRed (control) or DsRed p150^glued^ 217-548 for 24 h. Cells were then fixed with PFA and stained for gamma tubulin. Images represent centrosomes in cells from the same coverslip taken with the same camera settings. Scale bar, 1 μm. (**b**) Quantification of (a). GFP-CCDC66 fluorescence intensities were measured in a 2.5 μm^2^ circular area around the centrosome from two independent experiments. Levels are normalized to the mean of the control group (=1). n = 50 cells for each group. t-test was used for statistical analysis. Error bars, SEM: DsRed = 0.12, p150^glued^ CC1 = 0.07. (**c**) Effect of inhibiting molecular motor activity on CCDC66 level at the centrosome. RPE1-CCDC66 cells were treated with 2 mM AMP-PNP for 10 min. Cells were then fixed and stained for GFP, PCM1 and gamma tubulin. Scale bar, 1 μm. (**d**) Quantification of (a). GFP-CCDC66 fluorescence intensities were measured in a 2.5 μm^2^ circular area around the centrosome from two independent experiments. Levels are normalized to the mean of the control group (=1). n = 50 cells for each group. t-test was used for statistical analysis. Error bars, SEM: control = 0.84, AMP-PNP = 0.01. (**e**) Effect of inhibiting molecular motor activity on CCDC66 dynamics at the centrosome. RPE1::GFP-CCDC66 cells were transfected with DsRed (control) or DsRed p150^glued^ 217-548 for 24 h and in parallel they cells were treated with 2 mM AMP-PNP for 10 min. 2.5 μm^2^ circular area around the centrosome marked by yellow dashed circle was photobleached and imaged for 250 seconds after photobleaching. Still images represent centrosomal GFP-CCDC66 signal at the indicated times. Scale bar, 1 μm. (**f**,**i**) Percentage of recovery graph from (c). Individual FRAP experiments from two independent experiments were fitted into one phase association curves. n = 8 for DsRed and n = 6 for p150^glued^ CC1 transfected cells per group. n = 10 for control and n = 7 for AMP-PNP treated cells per group. Half-time of and mobile pool were calculated using recovery data. (**g**, **j**) mobile pool of (d). Error bars, SEM: DsRed = 1.35, p150^glued^ CC1 = 1.38, control = 2.65, AMP-PNP = 2.34. (**h**,**k**) half-time analysis of (d). Error bars, SEM: DsRed = 1.41, p150^glued^ CC1 = 1.95, control = 2.23, AMP-PNP = 1.52.

The association of CCDC66 with the dynein/dynactin complex might be required for its centrosomal targeting and dynamics. To test this, we performed quantitative immunofluorescence and FRAP experiments in RPE1::GFP-CCDC66 cells inhibited for dynein activity by expressing p150^glued^-CC1, which prevents dynein-dynactin binding (53). Analogous to the perturbation of the microtubule cytoskeleton, there was a significant decrease (DsRed-p150^glued^-CC1 = 0.38 ± 0.07, p < 0.0001, n = 50 in total) in the centrosomal abundance of CCDC66 in dynein-inhibited cells relative to control cells (DsRed = 0.93 ± 0.11, n = 50 in total) (Fig. 4a,b). FRAP analysis of centrosomal GFP-CCDC66 in dynein-inhibited cells showed a significant but small decrease in the percentage of recovery (DsRed = 60.5% ± 1.35, n = 20, DsRed-p150^glued^-CC1 = 54.4% ± 1.38, p = 0.0024, n = 16 in total) and halftime (DsRed = 38.4 s ± 1.41, DsRed-p150^glued^-CC1 = 33.5 s ± 1.95, p = 0.030, n = 12 in total) (Figs 4e–h, S3A,B). Likewise, centrosomal abundance of CCDC66 significantly decreased upon 2 mM AMP-PNP treatment (Control = 1.00 ± 0.08; AMP-PNP = 0.26 ± 0.01, p < 0.0001, n = 50 in total) (Figs 4c,d, S3C,D), which at high concentrations inhibits the activity of both kinesin and dynein motors^37,57^. The halftime for centrosomal GFP-CCDC66 also decreased significantly in 2 mM AMP-PNP-treated cells (Control = 40.25% ± 2.31, n = 20; AMP-PNP = 23.5% ± 1.52, p = 0.0006 n = 14 in total), (Fig. 4i–k). These results indicate that molecular motors, in particular the activity of the dynein/dynactin-complex, is required for the dynamic localization of CCDC66 to the centrosome, likely through cooperation with microtubules.

### Ciliary CCDC66 is not dynamic and its ciliary recruitment is regulated by satellites

**Figure 5:**
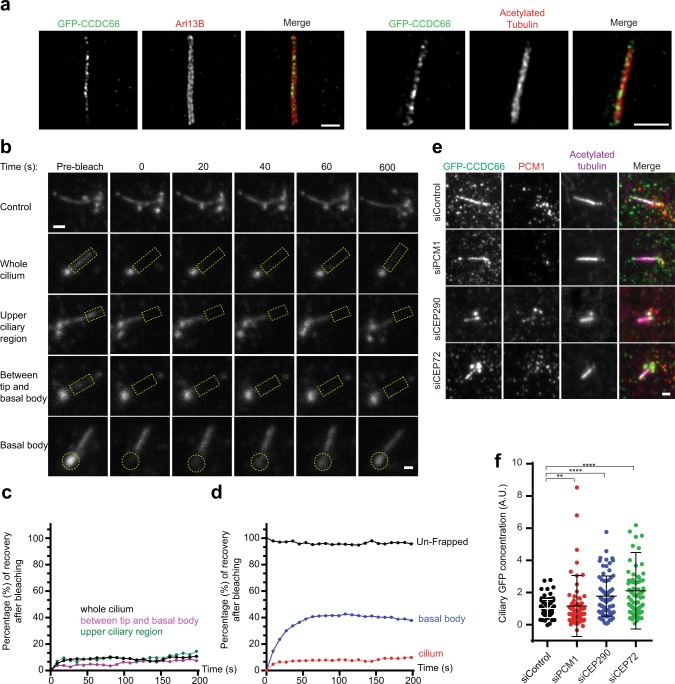
Ciliary CCDC66 is not dynamic and its recruitment is regulated by satellites. (**a**) STED analysis of ciliary CCDC66 localization. RPE1::GFP-CCDC66 cells were serum starved for 48 h, fixed with 4% PFA and stained for GFP, Arl13B (ciliary membrane marker) or acetylated tubulin (ciliary axoneme marker). Cells were imaged on a Leica TCS SP8 STED 3X confocal laser scanning microscope. Scale bar, 1 μm. (**b**) FRAP analysis of ciliary GFP CCDC66. RPE1::GFP-CCDC66 cells were serum starved for 48 h. Whole cilium, upper ciliary region or area between the tip and basal body indicated by yellow dashed rectangles was photobleached and cells were imaged for 250 seconds after photobleaching. Still images represent ciliary GFP-CCDC66 signal at indicated time points. Scale bar: 1 μm. (**c**,**d**) Percentage of recovery graph from (**b**). Individual FRAP experiments from two independent experiments (n In7 in total) were fitted into one phase association curves. (**e**) Effect of PCM1, CEP72 and CEP290 depletion on CCDC66 level at the cilia. RPE1::GFP-CCDC66 cells were transfected with control, PCM1, CEP290 or CEP72 siRNAs. 24 h after transfection, they were serum starved for 48 h. Cells were then fixed and stained for GFP, PCM1 and acetylated tubulin (cilia marker). Images represent cilia in cells from the same coverslip taken with the same camera settings. Scale bar, 1 μm. (**f**) Quantification of GFP-CCDC66 ciliary concentration from (**e**). GFP-CCDC66 fluorescence intensities were measured in the ciliary area defined by the acetylated tubulin ciliary marker from two independent experiments. Ciliary protein concentrations were determined by dividing fluorescence signal of the protein to the cilium length, which was quantified using acetylated tubulin staining. Levels are normalized to the mean of the control group (=1). n = 50 cells for each group. t-test was used for statistical analysis. Error bars, SEM: siControl = 0.06, siPCM1 = 0.21, siCEP290 = 0.14, siCCEP72 = 0.27.

In ciliated cells, CCDC66 localizes to the primary cilium in addition to centriolar satellites and the basal body. Time-lapse imaging of RPE1::GFP-CCDC66 confirmed its dynamic localization to the cilium upon serum starvation (Movie 1). To determine whether CCDC66 localizes to the ciliary axoneme or the membrane, we performed super-resolution microscopy using stimulated emission depletion (STED) analysis of CCDC66 relative to the axonemal marker acetylated tubulin and the ciliary membrane marker Arl13b in RPE1::GFP-CCDC66 cells. CCDC66 had a punctate localization pattern along the ciliary axoneme, but not at the ciliary membrane (Fig. 5a).

Through its localization at the axoneme, CCDC66 might function either as a structural component or as part of ciliary trafficking and signaling machineries, which would be reflected as static or dynamic ciliary pools, respectively. To test this, we quantified the dynamics of ciliary CCDC66 by performing FRAP analysis in RPE1::GFP-CCDC66 cells serum starved for 48 h. When the whole cilium was photobleached, the GFP-CCDC66 signal at the cilium did not recover even during 45 min of imaging after photobleaching (n = 7 in total) (Figs 5b,c, S4A). This indicates that there is no exchange between the cytoplasmic and the ciliary pools of CCDC66. When we photobleached the basal body and the cilium together, we observed a fast fluorescence recovery of 55.5% ± 2.20 with a half time of 67.2 s ± 5.41 at the basal body and again almost no recovery at the cilium (n = 16) (Figs 5b,d, S4D). To examine whether the low diffusional mobility of ciliary CCDC66 is due to its immobilization within the primary cilium, we performed half-cilium FRAP analysis in ciliated cells photobleached for the distal, middle or proximal parts of the cilium. Surprisingly, like whole-cilium FRAP experiments, there was almost no recovery in half cilium FRAP experiments (n = 7 in total) (Figs 5b,c, S4B,C). As a control, the same experiments were performed in RPE1 cells stably expressing GFP-fusion of the BBSome component BBS4. Analogous to CCDC66, BBS4 redistributes from centriolar satellites to the primary cilium upon serum starvation and functions in the ciliary exit of the G protein-coupled receptors (GPCRs)^49,58^. While most GFP-BBS4 did not recover in whole-cilium FRAP experiments, half cilium FRAPs revealed 41.6% ± 4.40 recovery of the bleached region with a half time of 48 s ± 7.88 from its unbleached ciliary pool (n = 8 in total) (Fig. S4E–L) This result shows that BBS4 is retained in the cilium once it is recruited and it is only dynamic within the primary cilium, analogous to the dynamic behavior of ciliary receptors such as the GPCR somatostatin receptor SSTR3 and serotonin receptor 6 HTR6^59^.

To test whether satellites regulate ciliary targeting of CCDC66 analogous to their function in its centrosomal targeting, we examined the ciliary levels and dynamics of GFP-CCDC66 in ciliated cells depleted for satellite components. The ciliary abundance of GFP-CCDC66 significantly increased in cells depleted for PCM1 (siPCM1 = 1.16 ± 0.21, p = 0.0018), CEP290 (1.77 ± 0.14, p < 0.0001) and CEP72 (2.1 ± 0.27, p < 0.0001) relative to control cells (siControl = 1.00 ± 0.06) (n = 100) (Fig. 5e,f). Because Cep72, Cep290 and PCM1 depletion compromises ciliogenesis efficiency, we only quantified CCDC66 levels in cells that ciliated (Fig. S5G). Loss of CEP72, CEP290 or PCM1 did not affect the percentage and halftime of recovery of ciliary CCDC66 in full-cilium FRAP experiments (n = 10 in total), supporting that the ciliary pool of CCDC66 does not exchange with its cytoplasmic pool after its ciliary recruitment (Fig. S5). Together, our results identify an inhibitory role for satellites in ciliary CCDC66 targeting, which is analogous to how they regulate its centrosomal targeting.

### Centriolar satellites exhibit both diffusive and directional motility

**Figure 6:**
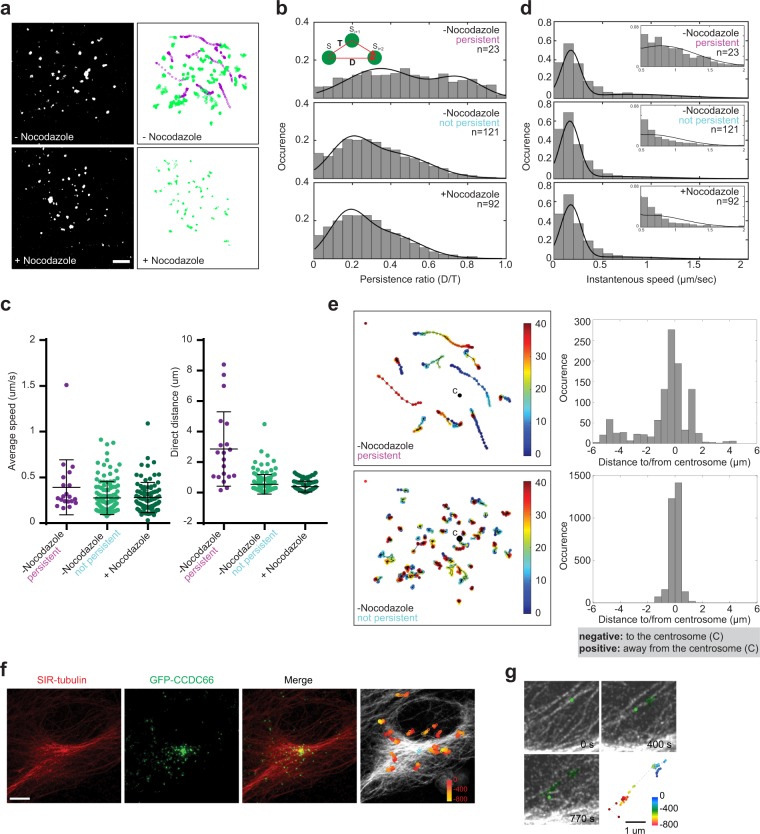
CCDC66-positive centriolar satellites exhibit both diffusive and microtubule-mediated directional motility. (**a**) Representative fluorescence images of satellites from time-lapse videos of RPE1::GFP-CCDC66 cells (top-left and bottom-left panels) and corresponding trajectories of satellites as a function of time (top-right and bottom-right panels). CCDC66-positive satellites were identified and tracked using the single-particle tracking algorithms detailed in Materials and Methods. Satellites were classified into persistent (magenta) and diffusive (green) motility groups using a persistence ratio cutoff value of 0.5. (**b**) The distribution of the persistence ratio (direct distance(D)/total distance(T)) was used to determine the different motility groups. Persistence histogram (gray bars) were fitted with a single or double Gaussian function (black line). (**c**) Average speed and direct distance of satellites. (**d**) The distribution of satellite instant speed. The changes at higher instant speed values were shown in the inset. (**e**) Time-colored trajectories of persistent (top-left) and diffusive (bottom-left) satellites and analysis of their directionality to the centrosome (right-hand graphs). Corresponding distribution of distance from/to centroids were plotted to determine the directed motility of satellites. Centroids were marked as C, indicating the localization of the centrosome. Negative values indicate movement towards the centrosome and positive values indicate movement away from the centrosome. (**f**) A representative fluorescence image of RPE1::GFP-CCDC66 cells stained with SIR-Tubulin. Images were overlaid to determine microtubule mediated movement of satellite that move persistently. The right-hand panel indicates the time dependent movement of satellites. (**g**) Representative satellite (green) exhibits bimodal motility by alternating between persistent and diffusive movements. Still images from Movie 6 at the indicated time points were shown. The bottom right-hand panel indicates the time dependent movement of the satellite.

The directed motility of a small fraction of satellites towards and away from the centrosome *in vitro* and *in vivo* has been used as evidence to suggest that satellites regulate protein targeting by physically trafficking proteins along microtubules^37^. This trafficking model predicts directional movement to or from centrosomes at a rate similar to that of motor-mediated transport along microtubules. To determine whether satellite dynamics is consistent with this model, we quantitatively assayed their dynamic behavior by combining time-lapse confocal imaging of interphase RPE1::GFP-CCDC66 cells with single particle tracking algorithms that we developed based on previous studies (described in Materials and Methods)^60,61^. We tracked a total of 144 satellites (n = 4) and a representative cell indicating the satellites with their associated trajectories is shown in Fig. 6a (Movie 2). This revealed different motility characteristics of satellites with varying degrees of persistence ratios (direct distance/total distance) where high persistence represents directional motility while low persistence represents random diffusion. We classified satellites into two groups - color-coded magenta and green - based on their average persistence ratio with a cutoff value of 0.5 (Fig. 6a), and quantified the average velocity and instant speeds of each group, as well as analyzed their directionality towards or from the centrosome.

The first group of satellites represented only about 14% of the total population (20 of 144 magenta trajectories in Fig. 6a). The histogram distribution of the persistence ratio of satellites showed two peaks with mean values of 0.35 and 0.79. This indicates that this group has bimodal motility, alternating between long range, directional motility (persistence > 0.5) and non-directional Brownian-type diffusion (persistence < 0.5) (Fig. 6b). The cutoff value for this classification was determined based on simulations of different motility modes ranging from low to high persistence (Fig. S6). Moreover, the calculation of their persistence ratio with a time difference of 8 ΔT and 12 ΔT both yielded similar distributions (Fig. S6). Their direct displacement value was 2.86 μm ± 0.55 and mean average velocity was 0.39 μm/sec ± 0.07 (mean ± SEM) (Fig. 6c). Given that the directional motility of satellites was interrupted with stationary pauses, the average speed values underestimated the speeds of long-range satellite movements. To determine whether speeds of satellites with long-range motility are compatible with the ones reported for molecular motors, we determined the instant speeds of satellites in consecutive frames. Only the persistent satellites (magenta-colored) had broad distribution at instant speeds higher than 0.5 μm/sec (Fig. 6d insets), which were consistent with the *in vivo* speeds reported for dynein and kinesins^62,63^. The rest of satellites had instant speeds slower than 0.5 μm/sec, representing the diffusive phases (Fig. 6d). One of the key predictions of the trafficking model is the directional motility of satellites towards or away from the centrosome. To test this, we quantified the difference between the distance of each satellite from the centrosome in consecutive frames (Fig. 6e). The occurrence of the negative and positive values supports directional movement towards the centrosome and away from the centrosome, respectively.

However, the majority of the satellites (124 of 144 or ≈86% of the total population, green trajectories in Fig. 6a) instead had a low persistent ratio with a single peak at 0.2 (Fig. 6b). This is indicative of short-range diffusive motility, and is inconsistent with a trafficking function for this population of satellites. They had a direct displacement value of 0.54 μm ± 0.06 and mean average velocity of 0.27 μm/sec ± 0.02 (mean ± SEM) (Fig. 6c). In contrast to the persistent satellites, they did not have a group of satellites with instant speeds higher than 0.5 μm/sec (Fig. 6d). This high occurrence of diffusive motility suggests that only a small fraction of satellites might mediate trafficking of cargo and the remaining population might mediate sequestration. In addition to satellite with diffusive and long-range motility, we observed fusion and splitting events between satellites and the centrosome, as well as between individual satellites (Fig. S7, Movies 3–5). This behavior is reminiscent of liquid-like structures^64^.

### The directed motility and random movement of satellites are mediated by microtubules

The linear directed motility exhibited by a small subset of satellites and the speed of their motility is reminiscent of molecular motor-mediated transport along microtubules. To test this, we investigated the effects of microtubule depolymerization on satellite dynamics. In nocodazole-treated cells, we did not observe any satellites with long, directional motility, confirming the requirement for microtubules for this type of motility (n = 92 satellites in 4 cells) (Movie 6). Instead, satellites had randomized Brownian-type movement, with a low persistent ratio of 0.20, direct displacement value of 0.39 μm ± 0.04 and mean average speed of 0.28 ± 0.02 μm/sec (mean ± SEM) (Fig. 6a–d). To visualize the movement of satellites along microtubules, we performed time-lapse imaging of RPE1::GFP-CCDC66 cells stained with SIR-tubulin that labels microtubules (Fig. 6f,g). We tracked a subset of CCDC66-positive satellites moving along microtubules interrupted with pauses and showed the color-coded trajectories of satellites with high persistence in superimposed images (Fig. 6f) (Movie 7). The orientation of the microtubules strongly aligned with the trajectory of satellites and some of them moved directionally towards or away from the centrosome (Fig. 6g). Together, our data confirms the requirement for microtubules for the persistent directional motility of satellites, which occurs in a subset of the population.

There is heterogeneity in the composition of individual satellites, and CCDC66 localizes only to a subset of satellites^15,43,65^. To investigate whether the dynamics of CCDC66-positive satellites are representative of all satellites in cells, we performed time-lapse imaging experiments in HeLa Kyoto cells stably expressing GFP-PCM1 from a bacterial artificial chromosome (BAC) transgene, which was recently used to study the function of FGFR1 oncogene partner (FOP) on satellite dynamics^66,67^. Representative satellites and their trajectories are shown in Fig. S8A (Movie 8). Analogous to CCDC66, the majority of PCM1-positive granules (113 of 150 or ≈76% of the total population, cyan trajectories) had diffusive motility with an average speed of 0.48 μm/sec ± 0.02, a direct displacement value of 0.75 μm ± 0.06 and a persistence ratio with a single peak at 0.2 (mean ± SEM) (Fig. S8B–D). A smaller fraction (37 of 150 or ≈24% of the total population, magenta trajectories) had a bimodal persistence ratio distribution of two peaks with mean values of 0.29 and 0.72 (Fig. S8B). Their mean average velocity was 0.74 μm/sec ± 0.06, direct displacement value was 2.9 μm ± 0.41 (mean ± SEM) (Fig. S8C), and a percentage of them moved towards or away from the centrosome (Fig. S8E). Upon nocodazole treatment, satellites exhibited Brownian-type diffusive motility, with a low persistent ratio of 0.18, direct displacement value of 0.73 μm ± 0.06 and mean average speed of 0.68 μm/sec ± 0.04 (n = 136) (mean ± SEM) (Fig. S8A–D) (Movie 9). Collectively, these results suggest that the heterogeneity in the composition of satellites is not reflected by changes in their dynamic behavior and in different cell types.

## Discussion

The dynamic alterations in the biogenesis, maintenance and function of the centrosome/cilium complex in response to physiological stimuli requires tight spatiotemporal control of the identity, quantity and modifications of the resident proteins. Defects in this regulation cause diseases like ciliopathies. In this study, we quantitatively assayed the dynamic behavior of CCDC66 at its different cellular locations, and elucidated the interplay between microtubules, motors, and satellites in regulating the targeting of CCDC66 to centrosomes and cilia. Since defective targeting of proteins to centrosomes and cilia contribute to disease phenotypes of ciliopathies, our results provide insight into cellular defects associated with retinal degeneration.

Comparative analysis of CCDC66 at its specific subcellular locations revealed different dynamics at these sites, likely indicative of the different functions of these compartments. Centrosomal CCDC66 was highly mobile with fast recovery time, which contrasts with the relatively immobile structural components of the centrosome, like centrin-2^68,69^. This dynamic exchange of CCDC66 between the centrosome and cytoplasm might be important for its ciliary and/or mitotic functions, which awaits further investigation. In contrast, ciliary CCDC66 was relatively immobile. Given that ciliary receptors and transport complexes studied so far including HTR6, SSTR3, IFT88 and BBS4 have fast ciliary dynamics^59^, we propose that CCDC66 may function as a structural component of the axoneme at the cilium, likely through its direct microtubule binding affinity. Interestingly, our STED analysis revealed that CCDC66 localized along the axoneme in a punctate pattern with irregular spacing, in contrast to the rather homogenous localization of acetylated tubulin, which may be linked to its function.

Although microtubules, molecular motors, and centriolar satellites all regulate protein targeting to the centrosomes, the interplay between them is poorly understood. We found that while microtubule organization and molecular motors were required for CCDC66 recruitment and kinetics at the centrosome, satellites inhibited it. We propose that microtubule-dependent CCDC66 targeting to the centrosome is mediated by two complementary mechanisms, which together will facilitate timely and rapid protein targeting. First, CCDC66 is actively transported to the centrosome along microtubules in a dynein-dependent manner, either by directly interacting with microtubules or indirectly by PCM1-negative protein complexes. Active transport mechanisms were previously reported for other centrosome proteins including CDK5RAP2^32,33^ and Par6alpha^34^. Second, microtubule ends and dynein complex components like p150^glued^ at the centrosome provide binding sides for CCDC66 through protein-protein interactions. This mechanism is supported by the interactions we identified for CCDC66 with microtubules and dynein complex components.

Previous studies identified requirements for centriolar satellites in centrosomal targeting of various proteins including centrin and pericentrin^37,39,40^. In contrast, we identified inhibitory functions for satellites in regulating centrosomal CCDC66 abundance and kinetics. This inhibition might be explained by their function as sequestration sites to limit CCDC66 centrosomal recruitment, which was previously also suggested but not tested, for regulation of the ubiquitin ligase Mib1^38^. Loss of satellites likely results in an increase in the cytoplasmic soluble pool of CCDC66, which will be available for incorporation into the centrosome. An alternative explanation could be satellite-mediated removal of CCDC66 from the centrosome. Although our quantitation of satellite dynamics identified satellites with persistent motility away from the centrosomes, these events were infrequent. We would also like to highlight that satellite components CEP72 and CEP290 both had inhibitory roles in CCDC66 centrosomal targeting, like PCM1. This is interesting as depletion of these proteins result in tighter clustering of satellites around the centrosome, which is a very different phenotype to the loss of satellites observed in PCM1-depleted cells^42^. Given that CCDC66 interacts with CEP72 and CEP290, this result might be explained by putative functions for CEP72 and CEP290 in mediating the interaction between PCM1 and CCDC66^43^.

The trafficking model for centriolar satellites was based on directed motility reported for a subset of satellites and changes in the centrosomal levels of various proteins in satellite-less cells^28,37–39,66^. The lack of quantitative analysis of satellite dynamics in cells in an unbiased way has made this model incomplete. In this study, we quantified the persistence, mean and instantaneous speeds and directionality of CCDC66- and PCM1-positivie satellites using live imaging combined with single particle tracking algorithms. Our analysis represents the first systematic quantitative characterization of the dynamic behavior of satellites and provides insight into their molecular mechanism of action. Supporting the trafficking function of satellites, we identified a subset of them to exhibit bimodal motility characterized by fast processive long-range motility towards or away from the centrosomes interrupted by pauses. However, these events were very infrequent, and the majority of satellites displayed short-range diffusive motility. Although previous studies described the bimodal motility for satellites qualitatively, the predominant diffusive behavior of satellites has not been reported^37,66,70^. This result has important implications for our understanding of satellite mechanisms. For example, satellite dynamics in specific contexts may be linked to specific cell types and functions or to different signals received by cells. Additionally, satellites might exert different modes of regulation for different centrosome and cilium proteins.

The higher ratio of non-directional diffusive satellites relative to directional ones is analogous to the dynamic behavior of other microtubule-associated cargoes such as autophagosomes, endosomes and peroxisomes^71^. However, only satellites cluster around the centrosome, and the molecular basis and functional significance of this clustering is not known. Various minus and plus end-directed molecular motors including BICD1, BICD2, DYNLL1, KIF7, KIF14, KIF20A were identified as components of satellites in proteomics studies^15,72^. The clustered satellite organization is likely a consequence of the minus end-directed motors dominating over plus end-directed motors during the tug of war between them. The binding affinities of satellites to different motors and the adaptors that mediate these interactions should be identified to elucidate how this tug of war ensures proximity of satellites to the centrosome.

Our findings reveal that satellites use different mechanisms to regulate protein targeting and that the interplay among these mechanisms change for different satellite-associated centrosome and cilium proteins (Fig. S9). Alongside active transport along microtubules, we here demonstrate a “sequestration-based mechanism” for regulating CCDC66 centrosomal and ciliary abundance. Despite the presence of a small population of CCDC66-positive satellites with persistent directional motility towards or away from the centrosome, the net effect of this trafficking did not dominate over sequestration. We speculate that satellites store protein around the centrosome in order to ensure their timely and rapid incorporation at the centrosome and cilia when required. Satellite-mediated storage and trafficking of the proteins might be advantageous for regulating protein targeting in response to physiological stimuli as it overcomes the limitations associated with lack of directionality in diffusion-based transport. An important next step in advancing this model will be the identification of the molecular basis of the release of proteins from the satellites, as well as the specific signals that initiates that release.

Finally, we anticipate our findings to provide important insight into our understanding of cellular compartmentalization through membrane-less structures. A major difference of satellites from most microtubule-associated cargoes is their membrane-less nature and consequential liquid-like behavior^73^. Importantly, satellites are regulators of the biogenesis and function of centrosomes, another membrane-less condensate^74^. Consistent with the liquid-like behavior of satellites, we observed fusion and splitting events between satellites and the centrosome, as well as between individual satellites (Fig. S7). These events were more frequent around the centrosome where satellites clustered and phase-separation might be a way for satellites to exchange material with the centrosome^64^. The trafficking and storage-mediated functions of satellites identify them as likely transit sites for centrosome and cilium proteins. This is analogous to the roles of the vesicular trafficking pathway in storing and targeting their cargoes. Satellites may represent a novel way of mediating communication between membrane-less compartments. Defining the full repertoire of satellite interactions, functions and mechanisms will be an important next step in testing this model.

## Materials and Methods

### Cell culture and transfection

Human telomerase immortalized retinal pigment epithelium cells (hTERT-RPE, ATCC, CRL-4000), RPE1::GFP-CCDC66^43^ and RPE1::LAP-BBS4 cells were cultured with Dulbecco’s modified Eagle’s Medium DMEM/F12 50/50 medium (Pan Biotech, Cat. # P04-41250) supplemented with 10% Fetal Bovine Serum (FBS, Life Technologies, Ref. # 10270-106, Lot # 42Q5283K) and 1% penicillin-streptomycin (Gibco, Cat. # 1540-122). Human embryonic kidney (HEK293T, ATCC, CRL-3216) cells were cultured with DMEM medium (Pan Biotech, Cat. # P04-03590) supplemented with 10% FBS and 1% penicillin-streptomycin. All cell lines were authenticated by Multiplex Cell Line Authentication (MCA) and were tested for mycoplasma by MycoAlert Mycoplasma Detection Kit (Lonza). RPE1 cells were transfected with the plasmids using Lipofectamine LTX according to the manufacturer’s instructions (Life Technologies, Ref. # 15338-100, Lot # 1995175). HEK293T cells were transfected with the plasmids using 1 μg/μl polyethylenimine, MW 25 kDa (PEI, Sigma-Aldrich, St. Louis, MO). For serum starvation experiments, cells were washed twice with PBS and incubated with DMEM/F12 50/50 supplemented with 0.5% FBS and 1% penicillin-streptomycin for the indicated times. For microtubule depolymerization and stabilization experiments, cells were treated with 10 μg/ml nocodazole (Sigma-Aldrich, Cat. #M1404) or 5 μM taxol (Sigma-Aldrich, Cat. #T7402) for 1 hour at 37 °C. For inhibition of dynein and kinesin activity, cells were treated with 2 µM unhydrolyzable ATP (AMP-PNP, Roche) for 10 minutes. For dynein inhibition experiments, DsRed-p150^glued^ (217–548) was expressed in cells for 24 h after transfection using Lipofectamine LTX.

### Plasmids and siRNA transfections

DsRed-p150^glued^ 217–548 was a gift from Trina Schroer (Addgene plasmid # 51146; http://n2t.net/addgene:51146; Addgene 51146)^75^. For depletion of PCM1, Cep72 and Cep290, previously characterized siRNAs were custom-ordered from Dharmacon. The siRNA sequences were GGGCUCUAAACGUGCCUCCUU for PCM1, GAUACUCGGUUUUUACGUAUUUU for Cep290 and UUGCAGAUCGCUGGACUUCAAUU for Cep72. Control siRNA#1 (Dharmacon, Cat. # D-001210-02-05) was used as control. Cells were seeded onto coverslips at 70% confluency and transfected with 50 nM of siRNA in two sequential transfections using Lipofectamine RNAiMAX (Life Technologies, Ref. # 13778-150, Lot # 2009103) in OPTI-MEM (Life Technologies, Cat. # 31985062) according to the manufacturer’s instructions. Depletion of proteins was confirmed 48 h, 72 h and 96 h after transfection by immunofluorescence and immunoblotting.

### Immunofluorescence, antibodies and microscopy

Cells were grown on coverslips, washed twice with PBS and fixed in either ice cold methanol at −20 °C for 10 minutes or 4% PFA in cytoskeletal buffer (10 mM PIPES, 3 mM MgCl_2_, 100 mM NaCl, 300 mM sucrose, pH 6.9) supplemented with 5 mM EGTA and 0.1% Triton X for 15 min at 37 °C^76^. After rehydration in PBS, cells were blocked with 3% BSA (Capricorn Scientific, Cat. # BSA-1T) in PBS + 0.1% Triton X-100 followed by incubation with primary antibodies in blocking solution for 1 hour at room temperature. Cells were washed three times with PBS and incubated with secondary antibodies and DAPI (Thermo Scientific, cat# D1306) at 1:2000 for 45 minutes at room temperature. Following three washes with PBS, cells were mounted using Mowiol mounting medium containing N-propyl gallate (Sigma-Aldrich). Primary antibodies used for immunofluorescence were rabbit anti Arl13b (17711-1-AP, Proteintech) at 1:100, mouse anti acetylated tubulin (clone 6-11B, 32270, Thermo Fischer) at 1:10000, mouse anti gamma tubulin (Sigma, clone GTU-88, T5326) at 1:1000, mouse anti GFP (3E6) 1:750, mouse anti alpha tubulin (Sigma, DM1A) at 1:1000 and mouse anti-PCM1 1:1000, rabbit anti CEP152 (Bethyl, A302-480A) at 1:500, mouse anti p150^glued^ (BD Biosciences, 610473) at 1:1000. Rabbit anti-PCM1 and anti-GFP antibodies were generated and used for immunofluorescence as previously described^65^. Secondary antibodies used for immunofluorescence experiments were AlexaFluor 488-, 568- or 633-coupled (Life Technologies) and they were used at 1:2000.

Time lapse live imaging was performed with Leica SP8 confocal microscope equipped with an incubation chamber. Asynchronous cells were imaged at 37 °C with 5% CO_2_ with a frequency of 0.375 seconds at a specific position for 100 frames in 512 × 512 pixel format using HC PL APO CS2 40 × 1.3 NA oil objective. For ciliogenesis videos, cells were incubated with 0.5% FBS in DMEM-12 just before imaging and imaged overnight with a frequency of 10 minutes per frame with 0.3 µm step size and 3.5 µm stack size in 1024 × 1024 pixel format. For centrosomal protein level quantifications, images were acquired with Leica DMi8 fluorescent microscope with a stack size of 8 µm and step size of 0.3 µm in 1024 × 1024 format using HC PL APO CS2 63 × 1.4 NA oil objective. Higher resolution images were taken by using HC PL APO CS2 63 × 1.4 NA oil objective with Leica SP8 confocal microscope. For STED analysis of ciliary GFP-CCDC66, images were acquired with Leica TCS SP8 STED 3X super resolution microscope, using HC PL APO 100 × 1.40 NA oil STED WHITE objective. All images were taken from single sections and deconvolved in Huygens software.

All centrosomal and ciliary level quantifications were done in Image J^77^. 2.5 µm^2^ or 17 µm^2^ region of interest (ROI) around the centrosome was determined using a centrosomal marker and the corresponding centrosomal signal was quantified. Cytoplasmic signal was subtracted from this value for every single cell. Statistical analysis was done by normalizing these values to their mean. Statistical significance was determined by Student’s t-test using Prism (GraphPad, La Jolla, CA). Ciliary length was measured using acetylated tubulin as the ciliary length marker. Ciliary protein concentration was determined by dividing ciliary protein signal by ciliary length. All values were normalized relative to the mean of the overall quantification (=1).

### Fluorescence recovery after photobleaching

FRAP experiments were performed with Leica SP8 confocal microscope using FRAP module. Cells were incubated with 10% FBS in DMEM-12 and kept at 37 °C with 5% CO_2_. ROI was set to 2.5 µm^2^ for centrosomal FRAP experiments. Since primary cilium length varied from cell to cell, a specific ROI was defined for each primary cilium. A z-stack of 4 µm with 0.5 µm step size was taken during pre and post bleaching for both centrosome and cilium FRAP experiments. Bleaching was done 2 iterations with 488 Argon laser with 100% power. Maximal projection of the files was performed in Leica LAS X software and analysis was done in ImageJ. Recovery graph quantifications, t-half and mobile pool quantifications were done using the equations as described^78^.

### Image analysis and tracking of CCDC66 and PCM1 satellites

The trajectories of GFP labelled satellites were determined by using a custom script written in MATLAB (R2017b, Mathworks, Natick, MA). Particle tracking code to determine the time-dependent positions of satellites was partially adapted from previous studies^60,61^. To remove point defects in images, all frames were initially convoluted by using a gaussian filter with a lower bound of 3 pixels and an upper bound of 40 pixels. A threshold filter by using the average pixel intensity was applied to determine the location of satellites. By comparing the intensity values of segmented satellites, time-dependent x and y coordinates of centroid positions were determined for trajectory analysis. To associate each satellite and to construct their trajectories, mean square displacement was computed to determine the most probable location of satellites in consecutive frames. A total of 8 frames with a maximum distance of 25 pixels was used as a recovery time of satellites if a satellite disappears between tracked frames. After satellites were linked, trajectories were displayed as an overlay on a fluorescent movie. An average of 140 satellites from four independent cells and a total of 50 frames were used to determine the motility dynamics of satellites. Total displacement, direct displacement, persistence ratio, average and instantaneous speed were quantified by using the coordinates of satellites from trajectory analysis.

### Analysis of satellite trajectories

Persistence ratio of satellites were analyzed as follows: Total (T) and direct (D) displacement were computed by using the coordinates of tracked satellites acquired at constant time intervals of 0.375 s. Direct displacement measured by an interval of 12 was divided by the cumulative displacement computed by an interval of 2 frames. Therefore, overestimation of persistence was avoided due to the position error in the trajectories of satellites. Persistence distribution did not significantly change when other intervals of 8 frames for direct distance were selected. Persistence histogram plots were constructed by using all detected satellites. To estimate the peak values in persistence distributions, data was fit by using two gaussian peaks. D/T ratio approaches to 1 (maximum value for completely linear motion) as satellites move persistently around the centrosome. Otherwise, low D/T ratios were expected as they move diffusively. A cutoff D/T ratio of 0.5 was used to identify persistent satellites in the movies. Computer simulation of different motility models were performed to determine expected persistence values. After selecting the range of rotating angle, trajectories were determined for low, medium and high persistence models. By using the coordinates from each simulation, persistence values were plotted and used to determine the average rations for a given model.

### Cell lysis and immunoblotting

Cells were lysed in 50 mM Tris (pH 7.6), 150 mM NaCI, 1% Triton X-100 and protease inhibitors for 30 min at 4 °C followed by centrifugation at 15.000 g for 15 min. The protein concentration of the resulting supernatants was determined with the Bradford solution (Bio-Rad Laboratories, CA, USA). For immunoblotting, equal quantities of cell extracts were resolved on SDS-PAGE gels, transferred onto nitrocellulose membranes, blocked with TBST in 5% milk for 1 hour at room temperature. Blots were incubated with primary antibodies diluted in 3% BSA in TBST overnight at 4 °C, washed with TBST three times for 10 minutes and blotted with secondary antibodies for 1 hour at room temperature. After washing blots with TBST three times for 10 minutes, they were visualized with the LI-COR Odyssey® Infrared Imaging System and software at 169 µm (LI-COR Biosciences). Primary antibodies used for immunoblotting were mouse anti-p150^glued^ (BD Biosciences, 610473) at 1:1000, rabbit anti BBS4 (Proteintech, 12766-1-AP) at 1:500, rabbit anti CCDC66 (Bethyl, A303-339A) at 1:2500, rabbit anti PCM1 (Proteintech, 19856-1-AP) at 1:500, rabbit anti CEP290 (Proteintech, 16268-1-AP) at 1:500, rabbit anti CEP72 (Proteintech, 22490-1-AP) at 1:500, mouse anti beta-actin (Proteintech, 60008-1-Ig) at 1:10000, mouse anti alpha-tubulin (Sigma, DM1A) at 1:5000. anti-PCM1 and anti-GFP antibodies were generated and used for immunobloting as previously described^65^. Secondary antibodies used for western blotting experiments were IRDye680- and IRDye 800-coupled and were used at 1:15000 (LI-COR Biosciences).

### Immunoprecipitation

HEK293T cells transfected with GFP or GFP-CCDC66 were resuspended in IP buffer (50 mM Tris-HCl pH 7.4, 260 mM NaCl, 2.27 mM KCl, 1.25 mM KH_2_PO_4_, 6.8 mM Na_2_HPO_4_, 1% NP-40) freshly supplemented with protease inhibitors (10 µg/ml Leupeptin, Pepstatin and Chymostatin, 1 mM PMSF, 10 µg/ml Aprotinin), tumbled at 4 °C for 45 min, and centrifuged at 13.000 R.P.M. for 15 minutes. Supernatants were added with 2 ug of control goat IgG or goat anti GFP antibody and tumbled at 4 °C for 2 hours. Meanwhile, SureBeads^TM^ Protein G Magnetic agarose beads (Bio-Rad) were washed with IP buffer three times and 30 ul (50% slurry) beads were added to supernatant and antibody mixes. Mixes were tumbled overnight. Next day, mixes were centrifuged at 1000 R.P.M for 1 minute and supernatants were saved as unbound fractions. Beads were washed with IP buffer two times and saved as bound fraction.

### *In vitro* microtubule pelleting assay

HEK293T cells transfected with GFP-CCDC66 were lysed in in BRB80 buffer (80 mM PIPES pH 6.8, 1 mM EGTA, 1 m M MgCl_2_) supplemented with protease inhibitors (10 µg/ml LPC, 1 mM PMSF and 10 µg/ml aprotinin) at 4 °C for 30 minutes and centrifuged at 13.000 R.P.M. for 15 minutes. Lysate was further cleared by centrifugation at 90.000 R.P.M. for 5 minutes at 4 °C with TLA100 rotor (Beckman). 2.5 ug cell lysate was brought to a final volume of 250 µl BRB80 supplemented with 1 mM GTP. Lysate was incubated with 25 µM taxol stepwise at 30 °C and with or without 0.5 mM AMP-PNP at 30 °C for another 30 minutes. Lysate was centrifuged at 55.000 R.P.M. for 10 minutes at 30 °C in a TLA100 rotor (Beckman) through a 125 ul 40% glycerol cushion prepared from BRB90 buffer supplemented with 1 mM GTP. Pellets were resuspended in the same final volume as the input and equivalent volumes of pellet and supernatant were separated by SDS-PAGE and processed by immunoblotting.

### Statistical tests

Statistical results, average and standard deviation values were computed and plotted by using Matlab and Prism. Two-tailed t-tests and one-way ANOVA (Tukey) tests were applied to compare measurements. Unless otherwise stated, *P < 0.05, **P < 0.01, ***P < 0.001, **** *P* < 0.0001 were chosen to determine the significance levels.

## Supplementary information

Supplementary Information

Movie 1

Movie 2

Movie 3

Movie 4

Movie 5

Movie 6

Movie 7

Movie 8

Movie 9
