# Supplementary material for: Centrosomal and ciliary targeting of CCDC66 requires cooperative action of centriolar satellites, microtubules and molecular motors
Source: Sci Rep. 2019 Oct 3;9:14250. doi: 10.1038/s41598-019-50530-4 (PMC6776500; doi:10.1038/s41598-019-50530-4)
Supplement: Supplementary file 1 — Supplementary Information [file 41598_2019_50530_MOESM1_ESM.pdf]

Supplementary Information

Centrosomal and ciliary targeting of CCDC66 requires cooperative action of centriolar satellites, microtubules and molecular motors

Deniz Conkar[1], Halil Bayraktar[2], Elif Nur Firat-Karalar[1]

[1]Department of Molecular Biology and Genetics, Koç University, Istanbul, Turkey 34450

[2]Department of Molecular Biology and Genetics, Istanbul Technical University, Istanbul, Turkey 34450

Corresponding Author:
Elif Nur Firat-Karalar
Department of Molecular Biology and Genetics
Koç University
Tel: (212) 3381677
Fax: (212) 3381559
E-mail: ekaralar@ku.edu.tr

Figure S1

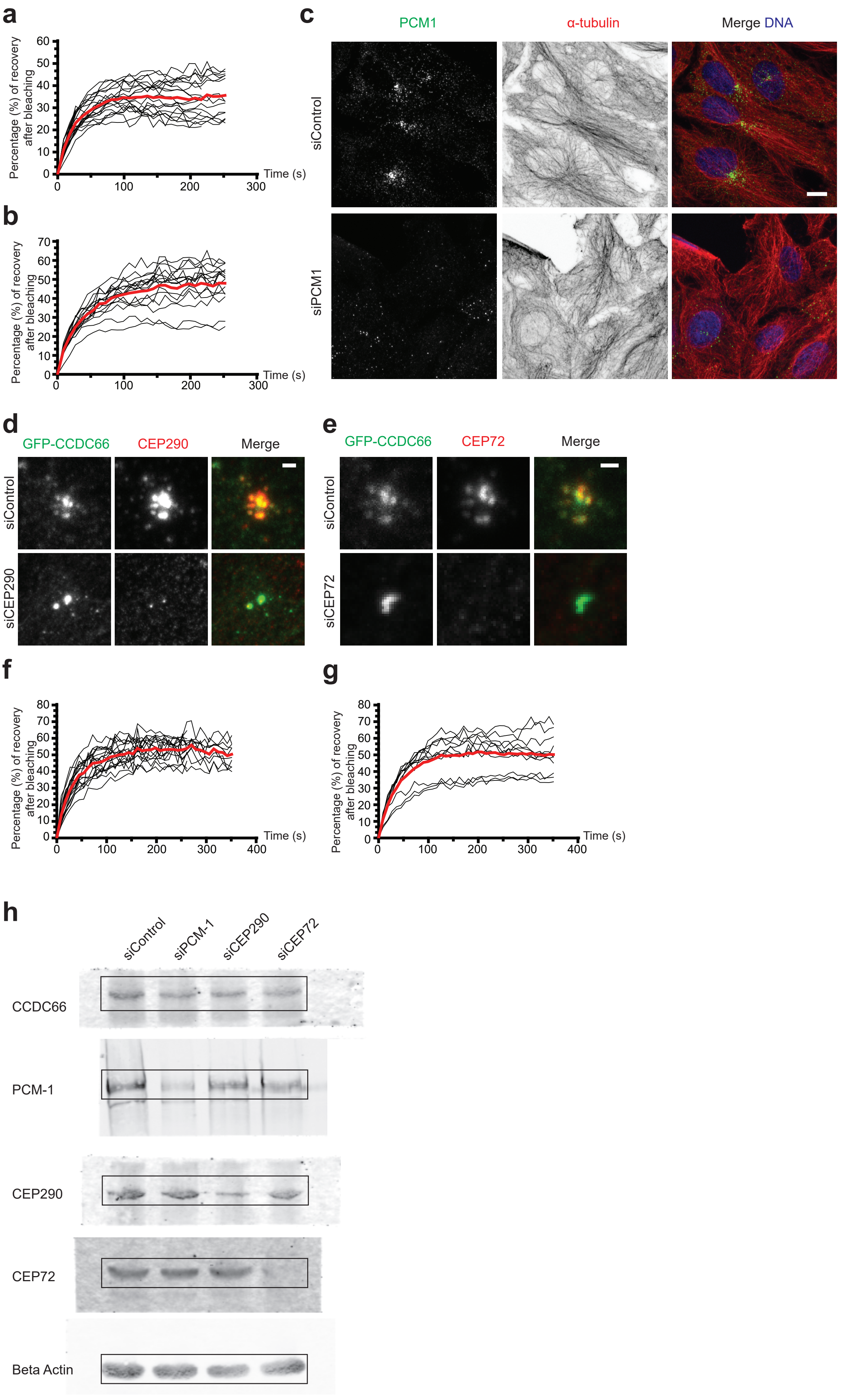
a
Percentage (%) of recovery after bleaching
Time (s)
b
Percentage (%) of recovery after bleaching
Time (s)
c
PCM1
α-tubulin
Merge DNA
siControl
siPCM1
d
GFP-CCDC66
CEP290
Merge
siControl
siCEP290
e
GFP-CCDC66
CEP72
Merge
siControl
siCEP72
f
Percentage (%) of recovery after bleaching
Time (s)
g
Percentage (%) of recovery after bleaching
Time (s)
h
siControl
siPCM-1
siCEP290
siCEP72
CCDC66
PCM-1
CEP290
CEP72
Beta Actin

Figure S2

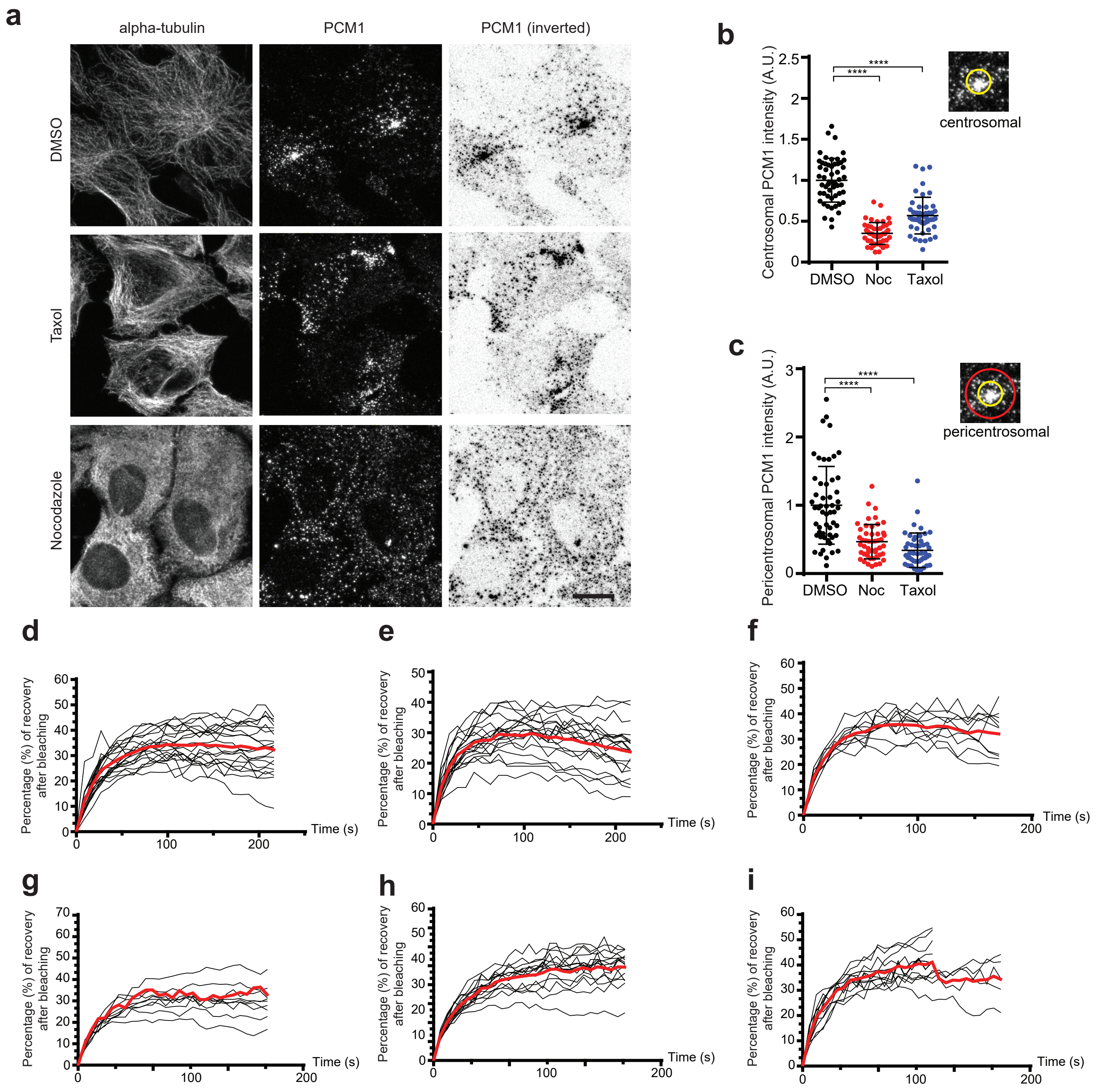

a
alpha-tubulin
PCM1
PCM1 (inverted)
DMSO
Taxol
Nocodazole
b
Centrosomal PCM1 intensity (A.U.)
****
****
DMSO
Noc
Taxol
centrosomal
c
Pericentrosomal PCM1 intensity (A.U.)
****
****
DMSO
Noc
Taxol
pericentrosomal
d
e
f
g
h
i
Percentage (%) of recovery after bleaching
Time (s)

Figure S3

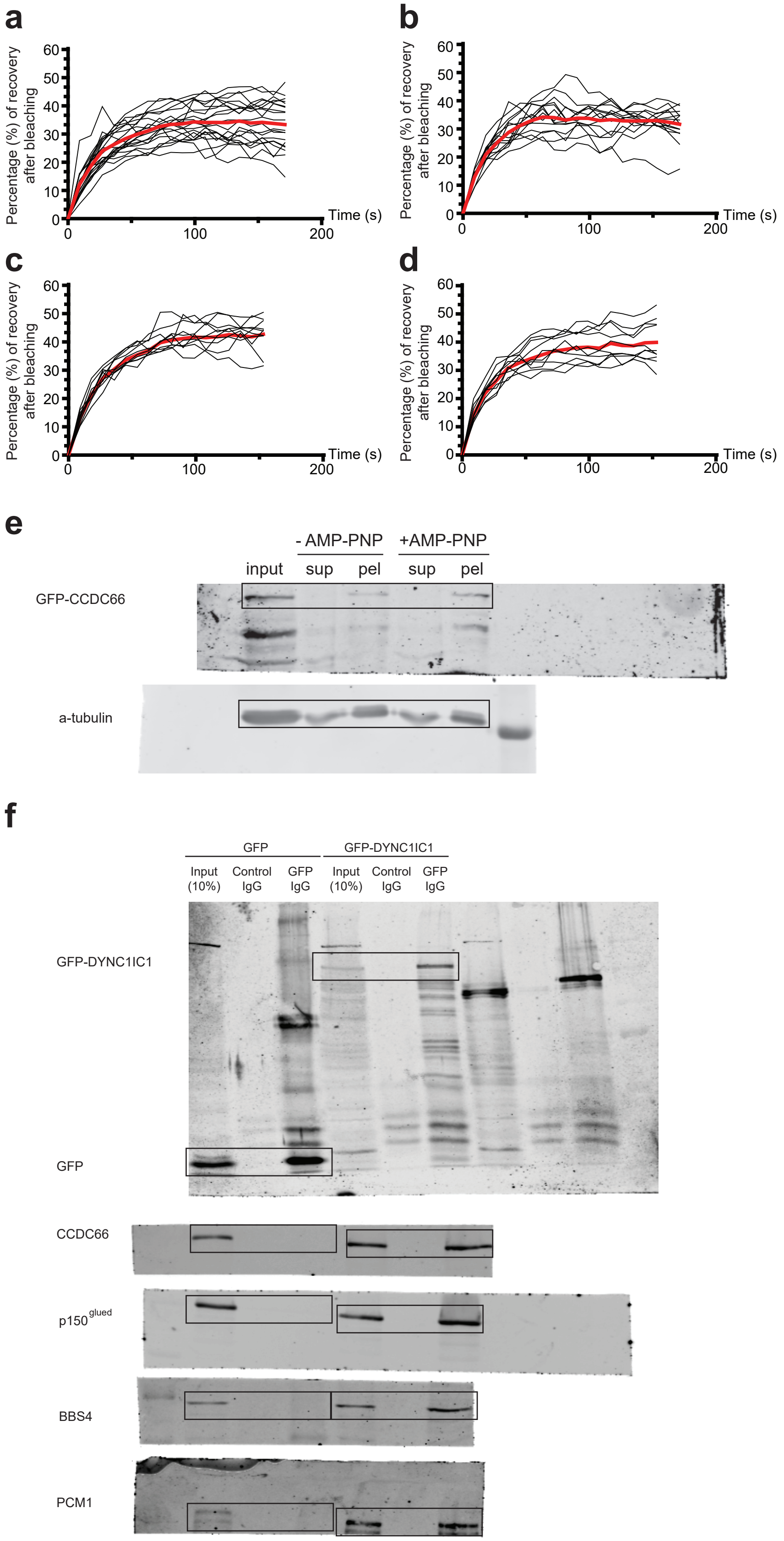

a
b
c
d
Percentage (%) of recovery after bleaching
Time (s)
0
10
20
30
40
50
60
100
200
e
- AMP-PNP
+AMP-PNP
input
sup
pel
sup
pel
GFP-CCDC66
a-tubulin
f
GFP
GFP-DYNC1IC1
Input (10%)
Control IgG
GFP IgG
Input (10%)
Control IgG
GFP IgG
GFP-DYNC1IC1
GFP
CCDC66
p150 glued
BBS4
PCM1

Figure S4

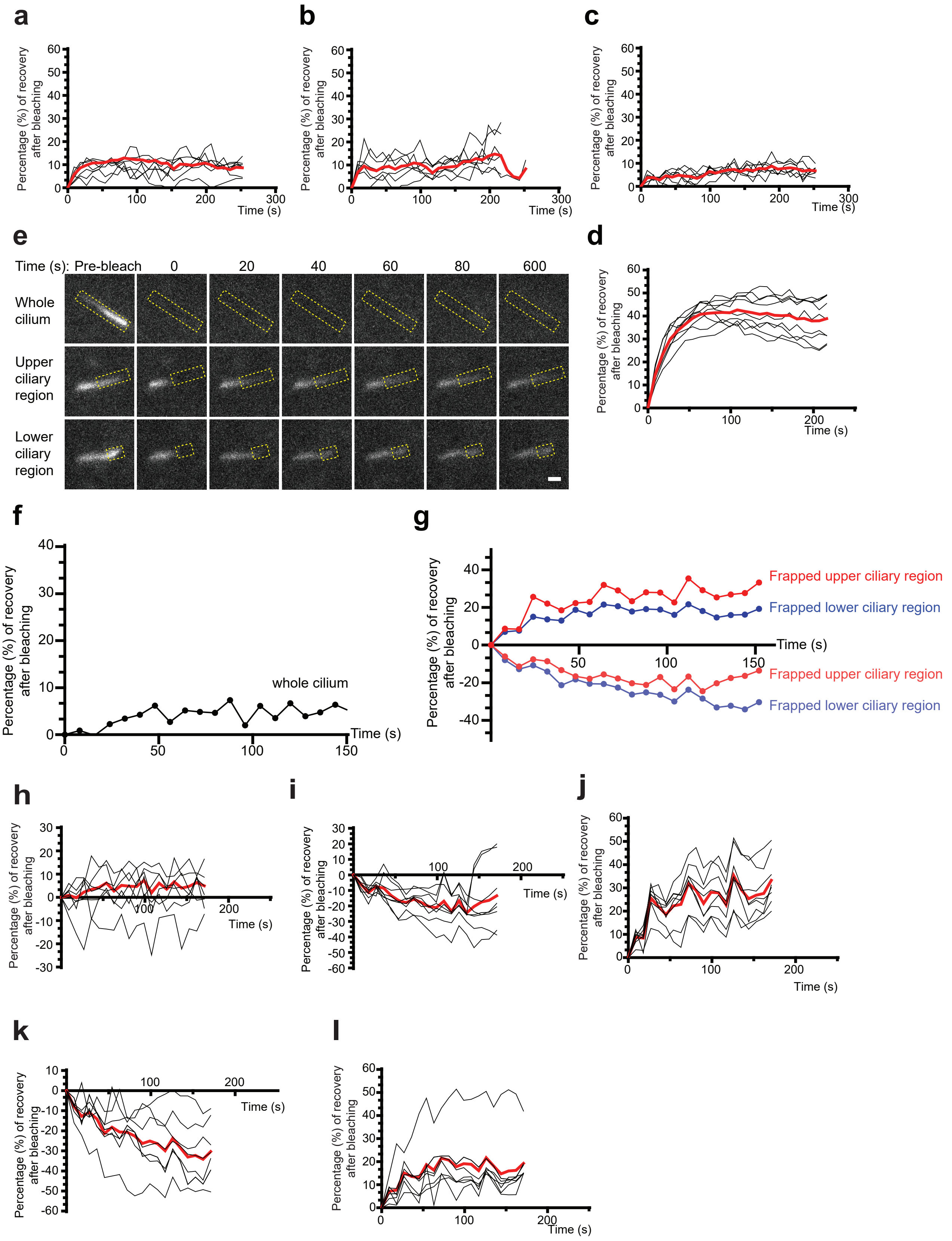

a
b
c
d
e
f
g
h
i
j
k
l
Percentage (%) of recovery after bleaching
Time (s)
Time (s): Pre-bleach
0
20
40
60
80
600
Whole cilium
Upper ciliary region
Lower ciliary region
whole cilium
Frapped upper ciliary region
Frapped lower ciliary region

Figure S5

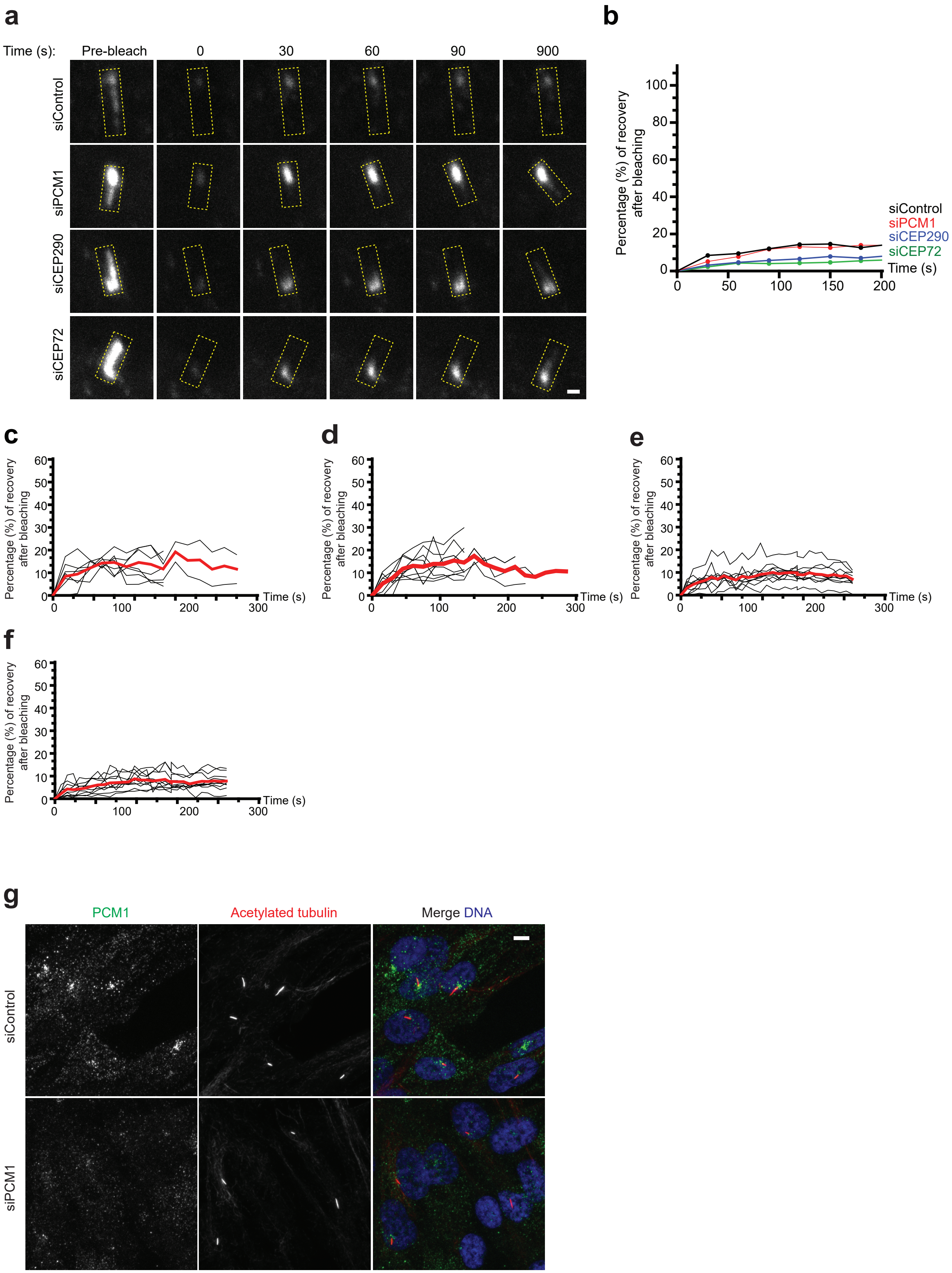
a
Time (s):
Pre-bleach
0
30
60
90
900
siControl
siPCM1
siCEP290
siCEP72
b
Percentage (%) of recovery after bleaching
siControl
siPCM1
siCEP290
siCEP72
Time (s)
c
d
e
f
Percentage (%) of recovery after bleaching
Time (s)
g
PCM1
Acetylated tubulin
Merge DNA
siControl
siPCM1

Figure S6

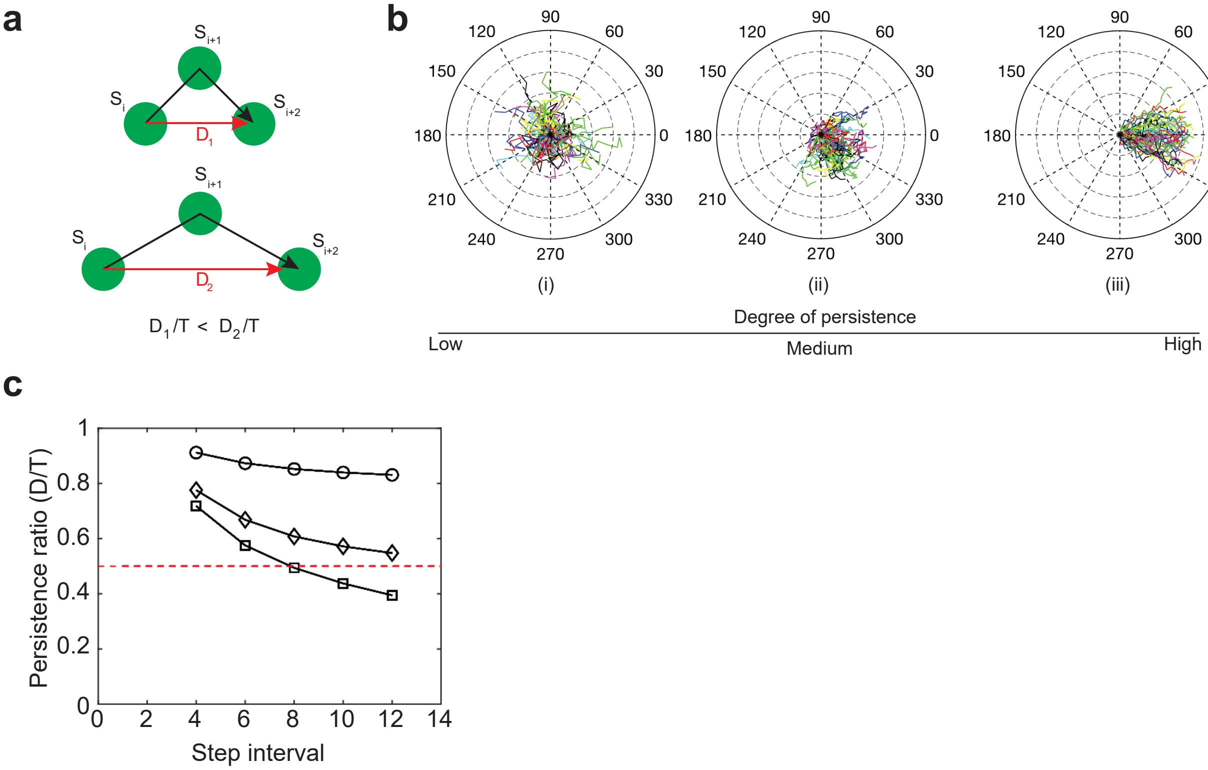

a
S i+1
S i
S i+2
D 1
D 1 /T < D 2 /T
b
90
60
30
0
330
300
270
240
210
180
150
120
(i)
(ii)
(iii)
Degree of persistence
Low
Medium
High
c
Persistence ratio (D/T)
Step interval

Figure S7

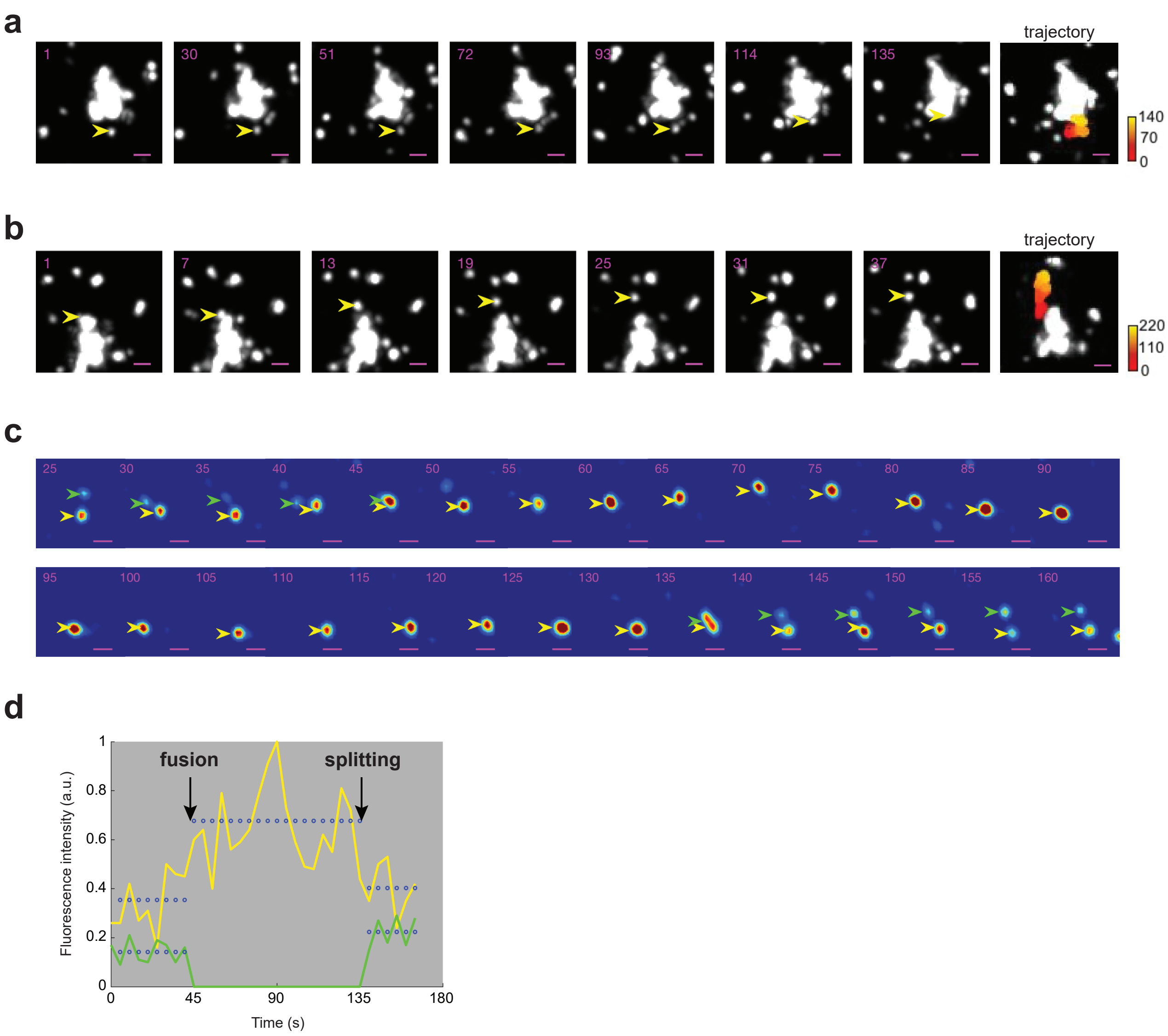
a
1
30
51
72
93
114
135
trajectory
140
70
0
b
1
7
13
19
25
31
37
trajectory
220
110
0
c
25
30
35
40
45
50
55
60
65
70
75
80
85
90
95
100
105
110
115
120
125
130
135
140
145
150
155
160
d
fusion
splitting
Fluorescence intensity (a.u.)
Time (s)
0
45
90
135
180
0.2
0.4
0.6
0.8
1

Figure S8

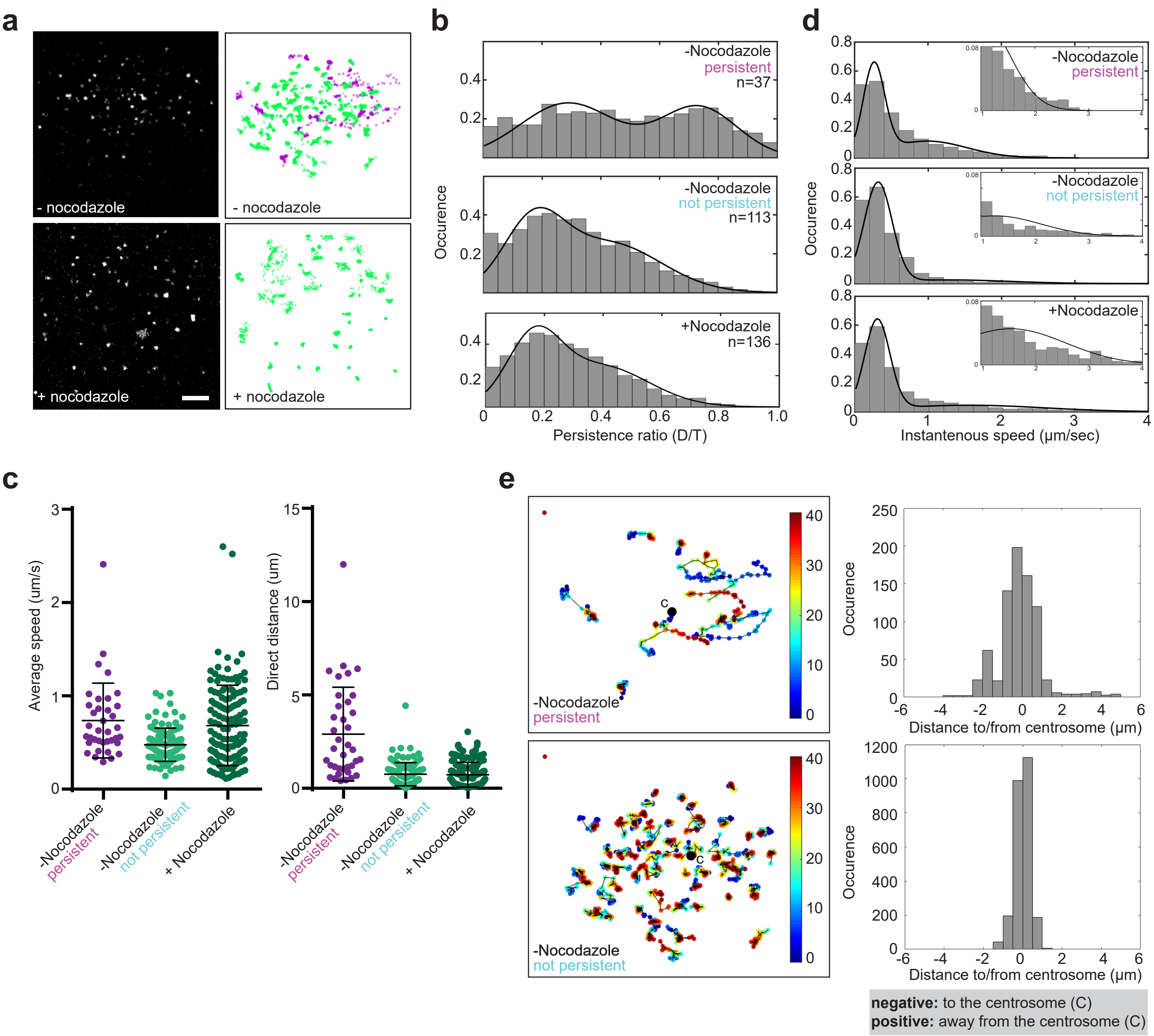

a
- nocodazole
- nocodazole
+ nocodazole
+ nocodazole
b
-Nocodazole
persistent
n=37
-Nocodazole
not persistent
n=113
+Nocodazole
n=136
Occurence
Persistence ratio (D/T)
d
-Nocodazole
persistent
-Nocodazole
not persistent
+Nocodazole
Occurence
Instantenous speed (µm/sec)
c
Average speed (um/s)
Direct distance (um)
-Nocodazole persistent
-Nocodazole not persistent
+ Nocodazole
e
-Nocodazole
persistent
-Nocodazole
not persistent
Occurence
Distance to/from centrosome (µm)
negative: to the centrosome (C)
positive: away from the centrosome (C)

Figure S9

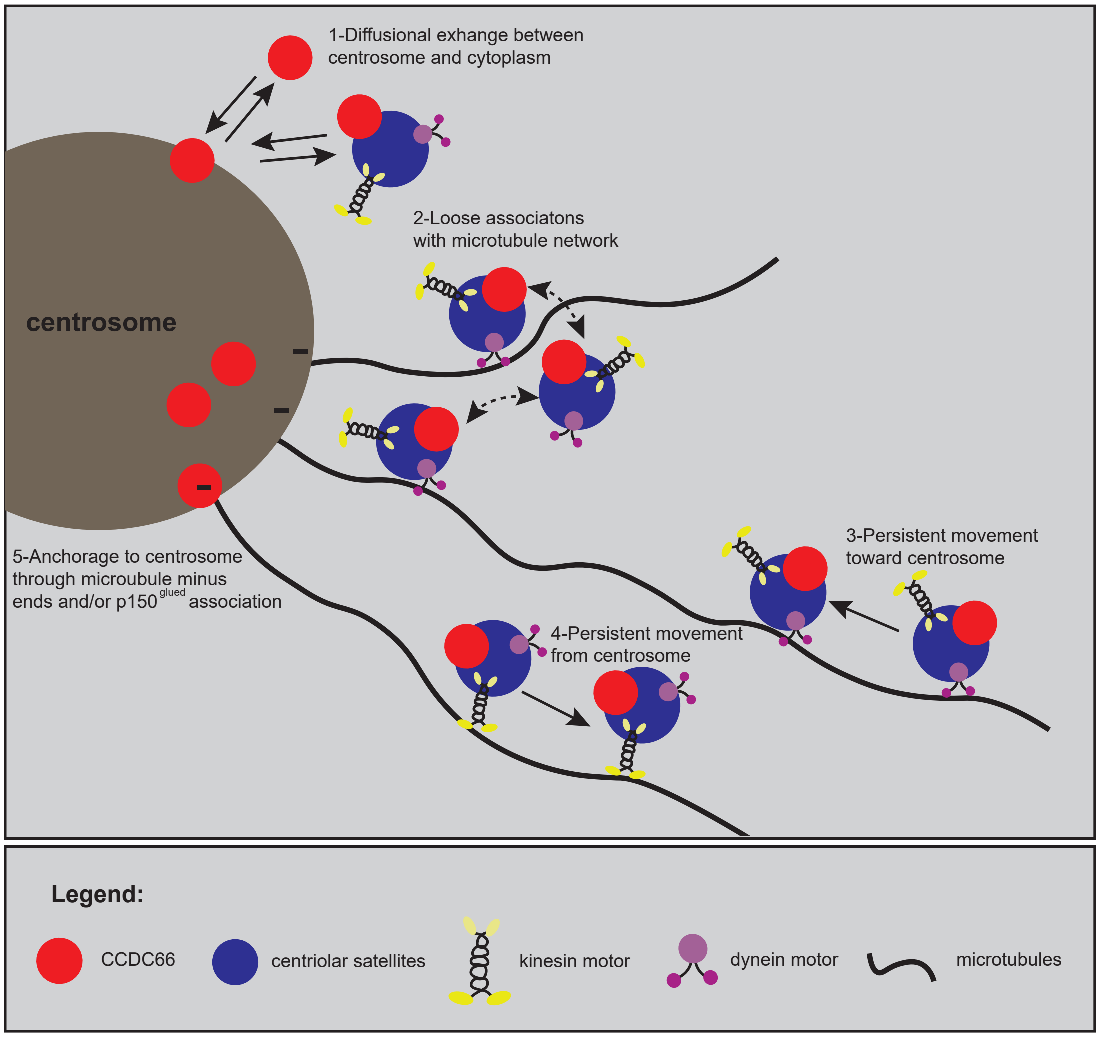
1-Diffusional exhange between centrosome and cytoplasm
2-Loose associatons with microtubule network
centrosome
3-Persistent movement toward centrosome
4-Persistent movement from centrosome
5-Anchorage to centrosome through microubule minus ends and/or p150glued association
Legend:
CCDC66
centriolar satellites
kinesin motor
dynein motor
microtubules

Supplementary Figure Legends

Figure S1

(a-b) Percentage of recovery graphs of individual FRAP experiments in control cells (a) and cells depleted for PCM1 (b). Individual FRAP experiments from two independent experiments were fitted into one phase association curves. n=8 for control and PCM1, depleted cells per group. Red curve represents the mean of all experiments.

(c) Effect of PCM1 depletion on microtubule network. RPE1 cells were transfected with control or PCM1 siRNAs. 48 h after transfection, cells were fixed and stained for PCM1 and α-tubulin. Images were taken with the same camera settings. Scale bar, 10 μm.

(d) RNAi-mediated depletion of CEP290 in RPE1::GFP-CCDC66 cells. RPE1-CCDC66 cells were transfected with control or CEP290 siRNA for 48 h. Cells were fixed and stained for GFP and CEP290. Scale bar, 1 μm.

(e) RNAi-mediated depletion of CEP72 in RPE1::GFP-CCDC66 cells. RPE1-CCDC66 cells were transfected with control or CEP72 siRNA for 48 h. Cells were fixed and stained for GFP and CEP72. Scale bar, 1 μm.

(f-g) Percentage of recovery graphs of individual FRAP experiments in control cells CEP290 (f) and Cep72 (g). Individual FRAP experiments from two independent experiments were fitted into one phase association curves. n=8 for CEP290 and n=6 for CEP72 depleted cells per group. Red curve represents the mean of all experiments.

(h) Uncropped and unmodified versions of immunoblots from Figure 1c. Brightness and exposure are adjusted in ImageJ.

Figure S2

(a) Effect of microtubule depolymerization and stabilization on PCM1 distribution. RPE1 cells were treated with 0.1% DMSO, 5 μg/ml nocodazole or 5 μM taxol for 1 h. Cells were the fixed with and stained for PCM1 and alpha-tubulin. Scale bar, 5 μm.

(b) Quantification of (a). PCM1 centrosomal fluorescence intensities were measured in a 2.5 μm^2 circular area around the centrosome from two independent experiments. Levels are normalized to the mean of the control group (=1). n=50 cells for each group. t-test was used for statistical analysis. Error bars, SEM: DMSO= 0.04, nocodazole=0.02, taxol=0.03.

(c) Quantification of (a). PCM1 pericentrosomal fluorescence intensities were measured by subtracting the fluorescence intensity of 17 μm^2 circular area from the 2.5 μm^2 circular area around the centrosome. Levels are normalized to the mean of the control group (=1). n=50 cells for each group. t-test was used for statistical analysis. Error bars, SEM: DMSO= 0.08, nocodazole=0.04, taxol=0.04.

(d-f) Percentage of recovery graphs of individual FRAP experiments in cells treated with 0.1% DMSO (d), 5 µg/ml nocodazole (e) or 5 µM taxol (f) for 1 h. FRAP experiments were performed after treatment. Individual FRAP experiments from two independent experiments were fitted into one phase association curves. n=10 for DMSO, n=9 for nocodazole and n=5 for taxol treated cells per group. Red curve represents the mean of all experiments.

(g-i) Percentage of recovery graphs of individual FRAP experiments in control depleted cells (g), cells depleted for PCM1 for 48 h (h) and in cells depleted for PCM1 for 48 h followed by nocodazole treatment for 1 h (i). Individual FRAP experiments from two independent experiments were fitted into one phase association curves. n=12 for control depleted, n=12 for PCM1 depleted and n=10 for PCM1 depleted and nocodazole treated cells per group. Red curve represents the mean of all experiments.

Figure S3

(a, b) Percentage of recovery graphs of individual FRAP experiments in control cells transfected with DsRed (a) and in cells inhibited for dynein activity after transfection with DsRed $p150^{glued}$ 217-548 for 24 h (b). Individual FRAP experiments from two independent experiments were fitted into one phase association curves. n=8 for DsRed and n=6 for $p150^{glued}$ CC1 cells per group. Red curve represents the mean of all experiments.

(c,d) Percentage of recovery graphs of individual FRAP experiments in control cells (c) and in cells treated with 2 mM AMP-PNP for 10 minutes. Individual FRAP experiments from two independent experiments were fitted into one phase association curves. n=10 for control and n=7 for AMP-PNP treated cells per group. Red curve represents the mean of all experiments.

(e,f) Uncropped and unmodified versions of immunoblots from Figure 3a (e) and 3c (f). Brightness and exposure are adjusted in ImageJ.

Figure S4

(a-d) Percentage of recovery graphs of individual ciliary GFP-CCDC66 FRAP experiments of whole cilium (a), upper ciliary region (b), between tip and basal body (c) and on basal body (d). Cells were imaged for 250 seconds post-bleaching. Red curve represents the mean of all experiments.

(e) FRAP analysis of ciliary GFP-BBS4. RPE1::GFP-BBS4 cells were serum starved for 48 h. Whole cilium, upper and lower ciliary regions indicated by yellow dashed rectangles were photobleached and cells were imaged for 150 seconds post-bleaching. Still images represent the ciliary GFP-BBS4 signal in indicated time points. Scale bar: 1 μm.

(f) Percentage of recovery graph of the whole cilium FRAP from (a). Percentage of recovery graphs from two independent experiments were fitted into one phase association curves. n=4 per group.

(g) Percentage of recovery graph of the upper and lower ciliary FRAPs from (a). Percentage of recovery graphs from two independent experiments were fitted into one phase association curves. n=4 cells per group. Curves in the positive y axis represent frapped upper (red) and ciliary (blue) regions. Curves in the negative y axis represent unbleached (unfrapped) upper (red) and ciliary (blue) regions.

(h-l) Percentage of recovery graphs of individual ciliary GFP-BBS4 FRAP experiments from whole cilium (h), upper ciliary region (j) and lower ciliary region (l). Signal from unbleached half of the cilium for upper ciliary (i) and lower ciliary (k) was quantified and plotted as well. Cells were imaged up to 200 seconds post-bleaching. Red curve represents the mean of all experiments.

Figure S5

(a) FRAP analysis of ciliary GFP-CCDC66 in cells depleted for PCM1, CEP290 or CEP72 . RPE1::GFP-CCDC66 cells were transfected with control, PCM1, CEP290 or CEP72 siRNAs. 24 h after transfection, they were serum starved for 48 h. Whole cilium and basal body regions indicated by yellow dashed rectangles were photobleached and

cells were imaged for 250 seconds post-bleaching. Still images represent ciliary GFP-CCDC66 signal at indicated time points. Scale bar: 1 µm.

(b) Percentage of recovery graph of different ciliary regions from (a). Ciliary signals were quantified excluding the basal body area. Percentage of recovery graphs from two independent experiments were fitted into one phase association curves. n=3 for control, n=5 for PCM1, CEP290 and CEP72 depleted cells per group.

(c-f) Percentage of recovery graphs of individual ciliary GFP-CCDC66 FRAP experiments from Control (e), PCM1 (f),CEP290 (g), and siCEP72 (h) depleted and serum starved cells. Cells were imaged up to 840 seconds post-bleaching. Red curve represents the mean of all experiments.

(g) Effect of PCM1 depletion on cilium formation. RPE1 cells were transfected with control or PCM1, CEP290 or CEP72 siRNAs. 24 h after transfection, they were serum starved for 48 h. Cells were then fixed and stained for PCM1 and acetylated tubulin (cilia marker). Images represent cilia in cells taken with the same camera settings. Scale bar, 5 µm.

Figure S6

(a) Schematic depiction of persistent movement by measuring direct and total distance of satellites. Higher values of direct distance results in persistent movement.

(b) Motility models that describe low, medium and high degree of persistent were shown in polar plots. All simulated trajectories were originated from origin.

(c) Computation of persistence ratio as a function of step interval for direct distance. A red horizontal line marks the cutoff value for persistent and diffusive motility.

Figure S7

(a) Time-lapse fluorescence images (1.43 s/frame) showing GFP-CCDC66 fused with the centrosome. Frame time (seconds) is shown at the top of each image. Time-colored trajectory of a satellite that approaches to the centrosome (right panel). Scale bar= 0.75 µm.

(b) Time-lapse fluorescence images (1.43 s/frame) showing GFP-CCDC66 splitted from the centrosome. Frame time (seconds) is shown at the top of each image. Time-colored trajectory of a satellite that moves away from the centrosome (right panel). Scale bar= 0.75 μm.

(c) Representative time-lapse images (5.0 s/frame) showing the fusion and splitting events between GFP-CCDC66-positive satellites (arrowheads). Colors denote the changes of their fluorescence intensity before and after fusion. Frame time (seconds) is shown at the top of each image. Scale bar= 0.3 μm.

(d) Quantification of fluorescence intensity changes of satellites shown in Fig. S7C. Fusion of satellites resulted in an increase of signal intensity at 45 s and remained at this level until the complex split at 135 s.

Figure S8

(a) Representative fluorescence images of satellites from time-lapse videos of HeLa::GFP-PCM1 cells (top-left and bottom-left panels) and corresponding trajectories of satellites as a function of time (top-right and bottom-right panels). PCM1-positive satellites were identified and tracked using the single-particle tracking algorithms detailed in Materials and Methods. Satellites were classified into persistent (magenta) and diffusive (green) motility groups using a persistence ratio cutoff value of 0.5.

(b) The distribution of the persistence ratio was used to determine the different motility groups. Persistence histogram (gray bars) were fitted with a single or double Gaussian function (black line).

(c) Average speed and direct distance of satellites.

(d) The distribution of satellite instant speed. The changes at higher instant speed values were shown in the inset.

(e) Time-colored trajectories of persistent (top-left) and diffusive (bottom-left) satellites and analysis of their directionality to the centrosome (right-hand graphs). Corresponding distribution of distance from/to centroids were plotted to determine the directed motility of satellites. Centroids were marked as C, indicating the localization of the centrosome.

Negative values indicate movement towards the centrosome and positive values indicate movement away from the centrosome.

Figure S9

1- Diffusional exchange between centrosome and cytoplasm

Cytoplasmic CCDC66 exchanges with its centrosomal pool by passive diffusion. The exchange could either be with the soluble cytoplasmic pool or with the pool associated with centriolar satellites. In the latter case, satellites act as storage sites.

2- Association and dissociation with microtubules

Satellites associate and dissociate with microtubules and the switch between these states in response to signals might regulate the interplay between diffusive and persistent motilities.

3-4 Persistent, directional movement toward and away from centrosome

Centriolar satellites move on microtubules towards the centrosome in a dynein-dependent manner and away from the centrosome in a kinesin-dependent manner. This pool comprises a small percentage within the total satellite population.

5- Anchorage to centrosome through microtubule minus end and/or $p150^{glued}$ association

Microtubule ends and/or microtubule associated proteins like $p150^{glued}$ might provide binding sites for CCDC66 at the centrosome.

Legends for Movies

1.100% confluent RPE1::GFP-CCDC66 cells were imaged every 10 minutes for 10 hours. Imaging started right after serum starvation. Scale bar: 5 μm.

2. GFP-CCDC66 dynamics in the absence of nocodazole. Scale bar: 5 μm

3. Fusion of GFP-CCDC66 with the centrosome. Scale bar: 1.5 μm.

4. Splitting of GFP-CCDC66 from the centrosome. Scale bar: 1.5 μm.

5. Fusion and splitting events between GFP-CCDC66-positive satellites Scale bar: 1 μm.

6. GFP-CCDC66 dynamics in the presence of nocodazole. Scale bar: 5 μm

7. Tracking of GFP-CCDC66 on microtubules. Scale bar: 5 μm

8. GFP-PCM1 dynamics in the absence of nocodazole. Scale bar: 5 μm

9. GFP-PCM1 dynamics in the presence of nocodazole. Scale bar: 5 μm
